# Photocleavable Anionic Glues for Light-Responsive Nanoparticle Aggregates

**DOI:** 10.1021/jacs.2c11973

**Published:** 2023-02-09

**Authors:** Jinhua Wang, Tzuf Shay Peled, Rafal Klajn

**Affiliations:** Department of Organic Chemistry, Weizmann Institute of Science, Rehovot 76100, Israel

## Abstract

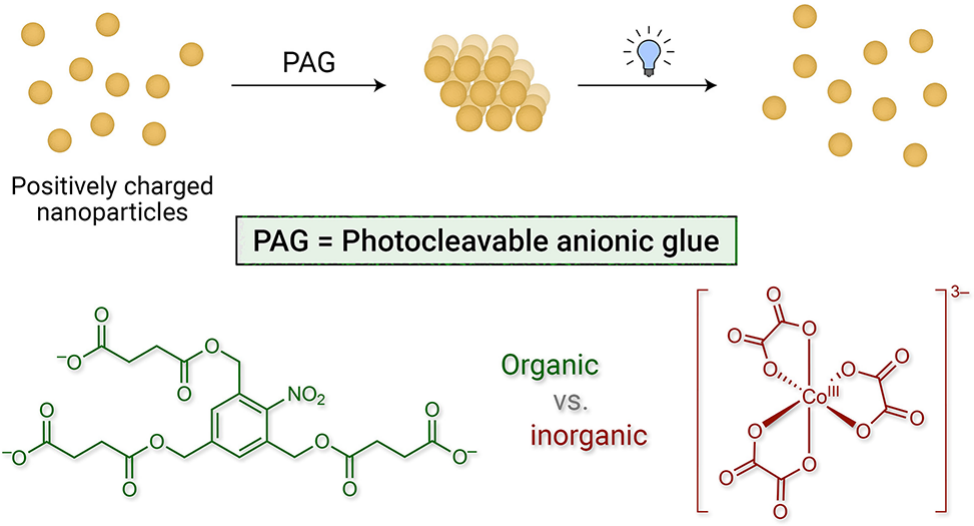

Integrating light-sensitive molecules within nanoparticle (NP) assemblies is an attractive approach to fabricate new photoresponsive nanomaterials. Here, we describe the concept of photocleavable anionic glue (PAG): small trianions capable of mediating interactions between (and inducing the aggregation of) cationic NPs by means of electrostatic interactions. Exposure to light converts PAGs into dianionic products incapable of maintaining the NPs in an assembled state, resulting in light-triggered disassembly of NP aggregates. To demonstrate the proof-of-concept, we work with an organic PAG incorporating the UV-cleavable *o*-nitrobenzyl moiety and an inorganic PAG, the photosensitive trioxalatocobaltate(III) complex, which absorbs light across the entire visible spectrum. Both PAGs were used to prepare either amorphous NP assemblies or regular superlattices with a long-range NP order. These NP aggregates disassembled rapidly upon light exposure for a specific time, which could be tuned by the incident light wavelength or the amount of PAG used. Selective excitation of the inorganic PAG in a system combining the two PAGs results in a photodecomposition product that deactivates the organic PAG, enabling nontrivial disassembly profiles under a single type of external stimulus.

## Introduction

Inorganic nanoparticles (NPs) exhibit a wide range of size-dependent properties (including optical, catalytic, and magnetic), which can be further controlled by the degree of NP aggregation and the NP–NP separation within the aggregates.^1^ For example, the optical,^2^ electronic,^3^ magnetic,^4^ and electric field enhancement^5^ properties of NPs have been manipulated by assembling NPs into aggregates with well-defined interparticle distances. It is particularly interesting to control the self-assembly of NPs using external stimuli, especially light. Several diverse strategies to achieve this goal have been developed.^6^ Most attention has been devoted to functionalizing the surface of NPs with photochromic ligands, such as azobenzenes^7−13^ and spiropyrans.^14−16^ Other approaches are based on phase transitions of NP-adsorbed thermoresponsive polymers,^17−19^ light-induced proton transfer between molecules in solution and NP-immobilized pH-sensitive ligands,^20−22^ as well as light-responsive molecules that bind to—and mediate interactions between—NPs upon exposure to light.^23−26^ These systems hold promise for applications in photoswitchable catalysis,^27^ reversible information storage,^20,28^ and controlled capture and release of small molecules from solution.^13,29^

However, other applications, such as controlled release or photolithography, call for irreversible light-induced transformations and do not require the (dis)assembly to be reversible. Compared with the light-induced reversible disassembly of NP aggregates, examples of irreversible disassembly are rare,^30,31^ and they are often accompanied by the coalescence of the particles’ inorganic cores.^32^ An attractive approach to design irreversible-disassembly systems is based on light-sensitive molecules that undergo irreversible transformation (e.g., cleavage of a covalent bond); such molecules include coumarin esters,^33−36^ benzophenone derivatives,^37^*meso*-substituted BODIPY dyes,^38−40^ truxillic acid derivatives,^31^ and others. Among them, *o*-nitrobenzyl derivatives^41^ are arguably the most widely used, with applications as diverse as 3D printing,^42^ photolithographic surface patterning,^43,44^ controlled drug release,^45^ operating molecular pumps,^46^ protection–deprotection strategies in peptide^47,48^ and organic^49^ synthesis, and light-triggered activation of biochemical processes^50^ (such as transcription^51−53^ and gene silencing^54^), among other applications.^55−62^

**Figure 1:**
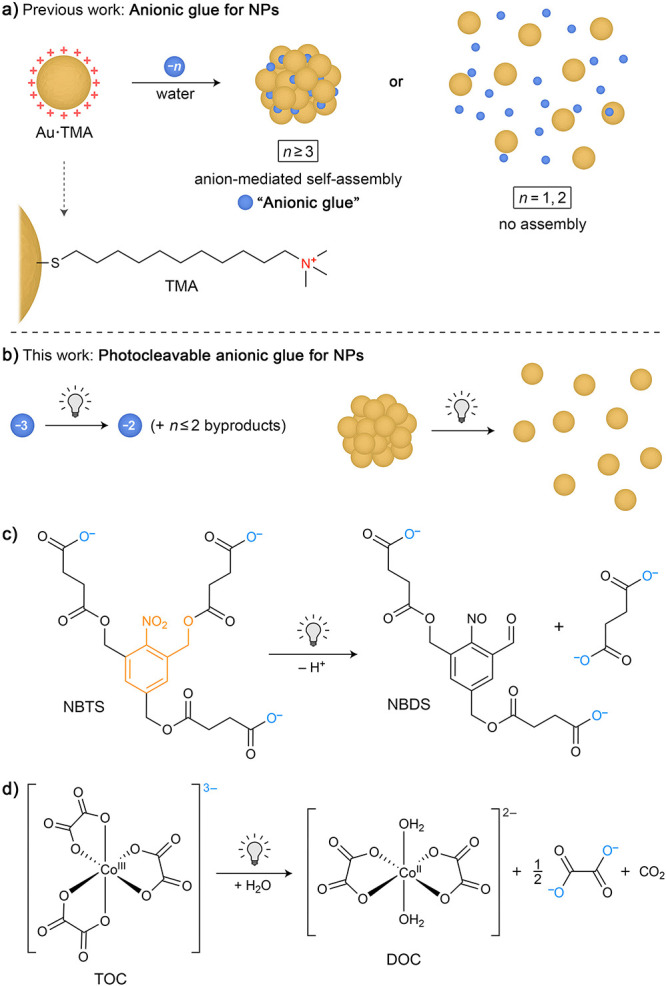
Concept of photocleavable anionic glue (PAG). (a) Self-assembly of positively charged, TMA-functionalized gold nanoparticles (NPs) mediated by anions having three or more negative charges (right: monoanions and dianions are incapable of mediating the self-assembly process). TMA = (11-mercaptoundecyl)-*N*,*N*,*N*-trimethylammonium; counterion = Br^–^. (b) Schematic representation of (left) the light-induced transformation of a trianion (PAG) into products having two or fewer negative charges and (right) the light-induced disassembly of NP aggregates held together by a PAG. (c) The structural formula and light-induced transformation of nitrobenzyltrisuccinate (NBTS; counterion = Na^+^) into nitrosobenzyldisuccinate (NBDS) and succinate dianions. (d) The structural formula and light-induced conversion of trioxalatocobaltate(III) (TOC; counterion = K^+^) into dioxalatocobaltate(II) (DOC) and byproducts having two or fewer negative charges.

We have recently reported the ability of small-molecule trianions (and anions bearing more than three negative charges) to mediate attractive interactions between cationic NPs in water.^63^ We found a sharp transition between the behavior of mono- and dianions (none of which induced aggregation of positively charged NPs) and that of higher oligoanions, which all acted as an “anionic glue” (Figure 1a). This behavior is explained by the Hardy–Schulze rule, which states that the coagulating potency of small oligoions toward oppositely charged colloids depends strongly on the oligoions’ charge.^64,65^ Therefore, we hypothesized that the light-induced transition of a trianion into a dianion (Figure 1b, left) might translate into light-triggered disassembly of NP aggregates.

Here, we introduce the concept of *photocleavable anionic glue* (PAG). PAGs are small light-sensitive anions capable of mediating attractive interactions between cationic NPs. Upon exposure to light, PAGs undergo decomposition into smaller molecules bearing two or fewer charges, incapable of supporting attractive interparticle interactions (i.e., unable to act as a “glue”). We demonstrate that anions as different as a newly synthesized, highly flexible trisuccinated *o*-nitrobenzyl derivative and a rigid Co(III) complex known for more than a century can act as PAGs and enable light-controlled disassembly of NP aggregates.

## Results and Discussion

### Self-Assembly of Cationic Gold NPs Mediated by an Organic PAG

To equip gold NPs with positive charges, we functionalized them with a thiol terminated with a positively charged trimethylammonium group^63,66^ (TMA in Figure 1a). Owing to their hydrophilic outer surface, these NPs can form colloidally stable suspensions in water, even at tens-of-millimolar concentrations (in terms of the concentration of Au atoms). However, the high density of positive charges facilitates the dissociation of TMA from the NPs and their slow sedimentation. To prevent this issue, we worked with NPs cofunctionalized with TMA and a shorter, electrically neutral ligand (1-hexanethiol) in a 9:1 molar ratio (which translated into a ∼4.6:1 ratio on the NPs; see Supporting Information, Section 3); these NPs combined high surface charge density with long-term colloidal stability. We refer to these NPs as Au·TMA.

Next, we sought to identify molecules that would behave as PAGs. First, we designed and synthesized a nitrobenzyl compound equipped with three succinate groups (nitrobenzyltrisuccinate, or NBTS). Upon exposure to UV (∼365 nm) light, the *o*-nitrobenzyl moiety’s C–O bond is cleaved to afford the corresponding *o*-substituted nitrosobenzene.^67−69^ For NBTS, this cleavage results in a nitrosobenzyldisuccinate (NBDS) and succinic acid dianion, each with a net charge of −2 (Figure 1c). NBTS was synthesized in three steps from commercially available starting materials; in the final, key step, 2,4,6-tri(hydroxymethyl)nitrobenzene was reacted with an excess of succinic anhydride (Supporting Information, Section 2.1).

**Figure 2:**
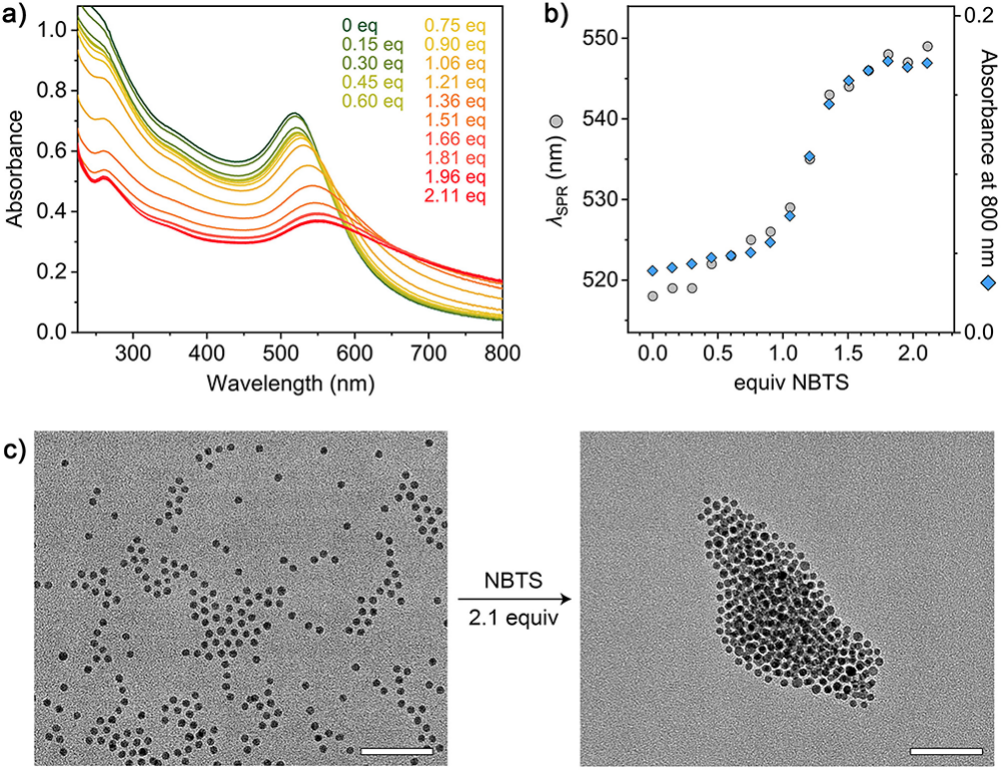
Self-assembly of positively charged nanoparticles (Au·TMA) mediated by NBTS. (a) A series of UV/vis absorption spectra recorded during the gradual addition of NBTS to a solution of 5.3 nm Au·TMA. The small absorption peak at ∼270 nm in the final spectra originates from the NBTS’ *o*-nitrobenzyl moiety (spectra not corrected for dilution; the increase in solution volume was <5% at the end of all the titration experiments). (b) Gradual increase in the LSPR’s wavelength of maximum absorption (λ_SPR_; gray markers) and the absorbance at 800 nm (blue markers) during the titration of 5.3 nm Au·TMA with NBTS (“equiv NBTS” denotes the molar ratio of negative charges (added as NBTS) to positive charges (the total number of NP-adsorbed TMA ligands in the titrated solution)). (c) Representative TEM images of TMA-functionalized 5.3 nm gold NPs before (left) and after (right) the addition of 2.1 equiv of NBTS (scale bars = 50 nm).

Figure 2a shows a series of UV/vis spectra of 5.3 ± 0.4 nm Au·TMA in the presence of increasing amounts of NBTS. The titration experiment was carried out in water at pH = 9 to ensure that most of the COOH groups were deprotonated. The initial spectrum features a pronounced absorption band centered at ∼520 nm; this band originates from the Au NPs’ surface plasmon resonance (SPR). When >1.0 equiv of NBTS (defined as the molar ratio of negative charges on NBTS to positive charges on NP-immobilized TMA) were added, the SPR band red-shifted to ∼550 nm and the absorbance at the high-wavelength region (800 nm) markedly increased (Figure 2a,b). These changes are indicative of NP aggregation. The S-shape of the titration curve is a consequence of the NP aggregation occurring most readily when the positive charges on the NPs are equalized by NBTS’ negative charges (similar to the precipitation titration of simple inorganic salts, such as AgCl). The continued addition of NBTS did not result in further changes in the UV/vis spectra.

The NBTS-mediated assembly of Au·TMA was confirmed by transmission electron microscopy (TEM), which showed that upon the addition of NBTS, the NPs formed amorphous aggregates in a near-quantitative fashion (i.e., practically no free NPs could be found; Figure 2c). To confirm that the assembly behavior does not depend on the NP size, we also synthesized 9.5 ± 0.5 nm Au NPs and decorated them with the same 9:1 TMA–hexanethiol mixture (which resulted in a ∼6.1:1 ratio on the NPs; Supporting Information, Section 3); titration with NBTS resulted in similar titration curves (Figure S5).

### Crystalline Assemblies of Cationic Gold NPs Mediated by an Organic PAG

**Figure 3:**
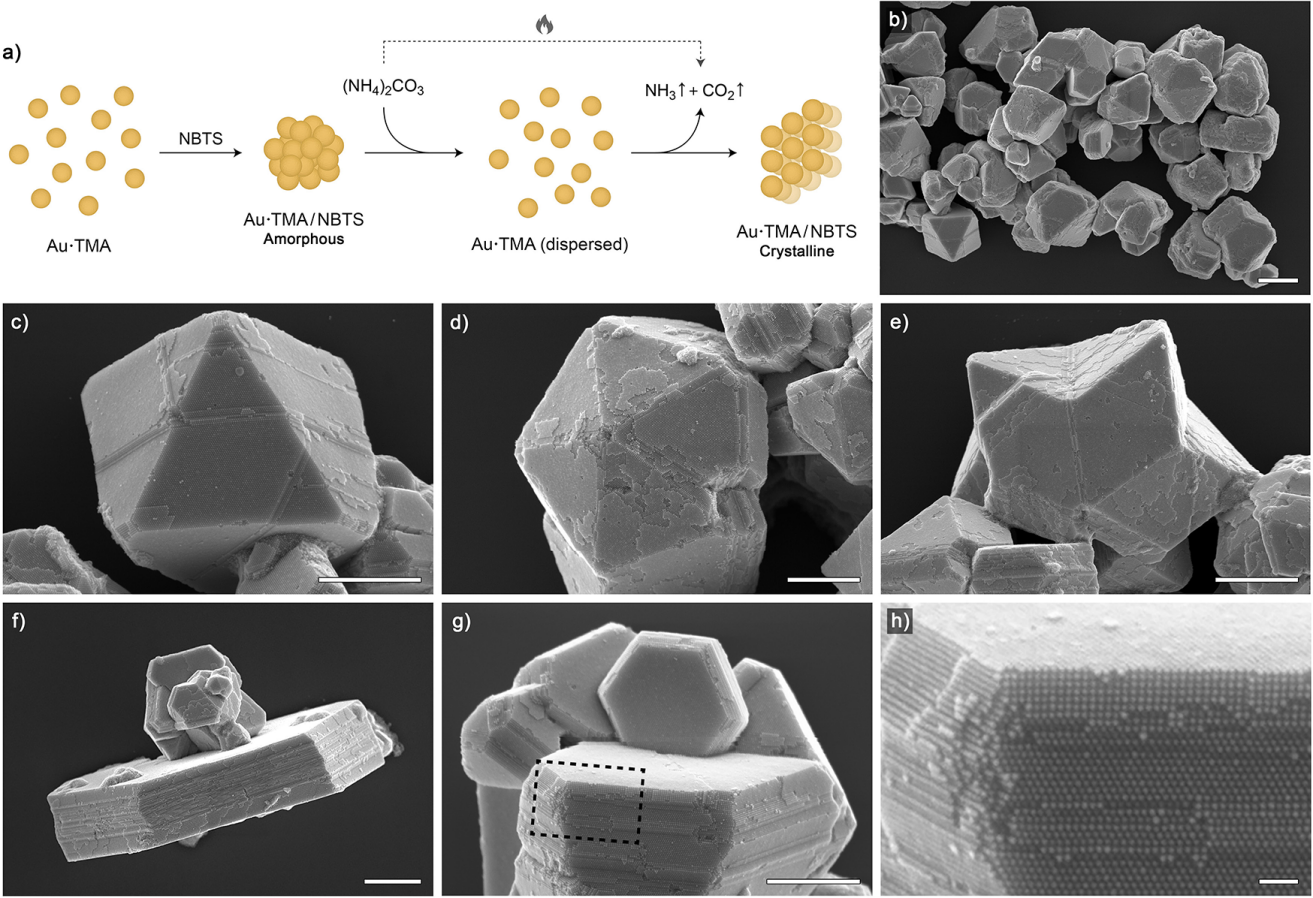
Colloidal crystallization of TMA-functionalized gold nanoparticles mediated by NBTS. (a) Schematic representation of (NH_4_)_2_CO_3_-induced transformation of amorphous Au·TMA/NBTS aggregates into crystalline ones. The flame symbol represents the exergonic nature of the ammonium carbonate decomposition reaction. (b–h) Representative scanning electron microscopy (SEM) images of colloidal crystals coassembled from 9.5 nm Au·TMA and NBTS (for additional images, see Figure S6). The micrograph in (h) is a magnified view of the region denoted by a dashed line in (g). The scale bars correspond to 1 μm in (b), (e), and (f), 500 nm in (c), (d), and (g), and 50 nm in (h).

We have recently described a method to convert amorphous aggregates of charged NPs into crystalline ones (NP superlattices).^63,70^ This method is based on temporarily increasing the ionic strength of the medium, thus screening the Coulombic interactions, and then gradually reintroducing them as the ionic strength spontaneously decreases. A convenient way to induce a spontaneous decrease of the ionic strength is to use volatile salts, such as ammonium carbonate (Figure 3a). The decomposition of (NH_4_)_2_CO_3_ into NH_3_, CO_2_, and H_2_O is a spontaneous (exergonic) reaction; an intriguing description of this process is that the energy released during the reaction is used to overcome the activation barrier separating the amorphous aggregates from the crystalline ones, thus converting the former into the latter. Here, we attempted to fabricate *light-sensitive NP superlattices*: crystalline assemblies of NPs incorporating the photocleavable NBTS as the ionic glue.

NBTS is significantly larger than the trianions previously reported to mediate the colloidal crystallization of Au·TMA (such as citrate and trimesate).^63^ However, the addition of a saturated solution of (NH_4_)_2_CO_3_ to amorphous Au·TMA/NBTS aggregates followed by its decomposition resulted in highly crystalline assemblies (Figure 3b–h), within which the NPs were held together by the NBTS PAG. Similar to the NP crystals reported before,^63^ Au·TMA/NBTS superlattices exhibited morphological features typical of the fcc structure,^71^ such as octahedra (Figure 3c), decagonal and star-shaped assemblies featuring 5-fold symmetries (Figure 3d,e), and hexagonal plates with abundant twin planes (Figure 3f–h). The fraction of the crystalline phase in the aggregates prepared from 9.5 nm Au·TMA (Figure 3) was consistently higher than in the 5.3 nm Au·TMA aggregates (Figure S7); in fact, practically all the 9.5 nm NPs assembled into crystalline aggregates. This difference can be explained by the combination of (i) the higher volume fraction of the “hard” pseudospherical Au component in the 9.5 nm NP aggregates and (ii) the lower size dispersity of the larger NPs (4.9% vs 7.3% for the 5.3 nm NPs).

### Light-Induced Disassembly of NP Aggregates via the Decomposition of an Organic PAG

**Figure 4:**
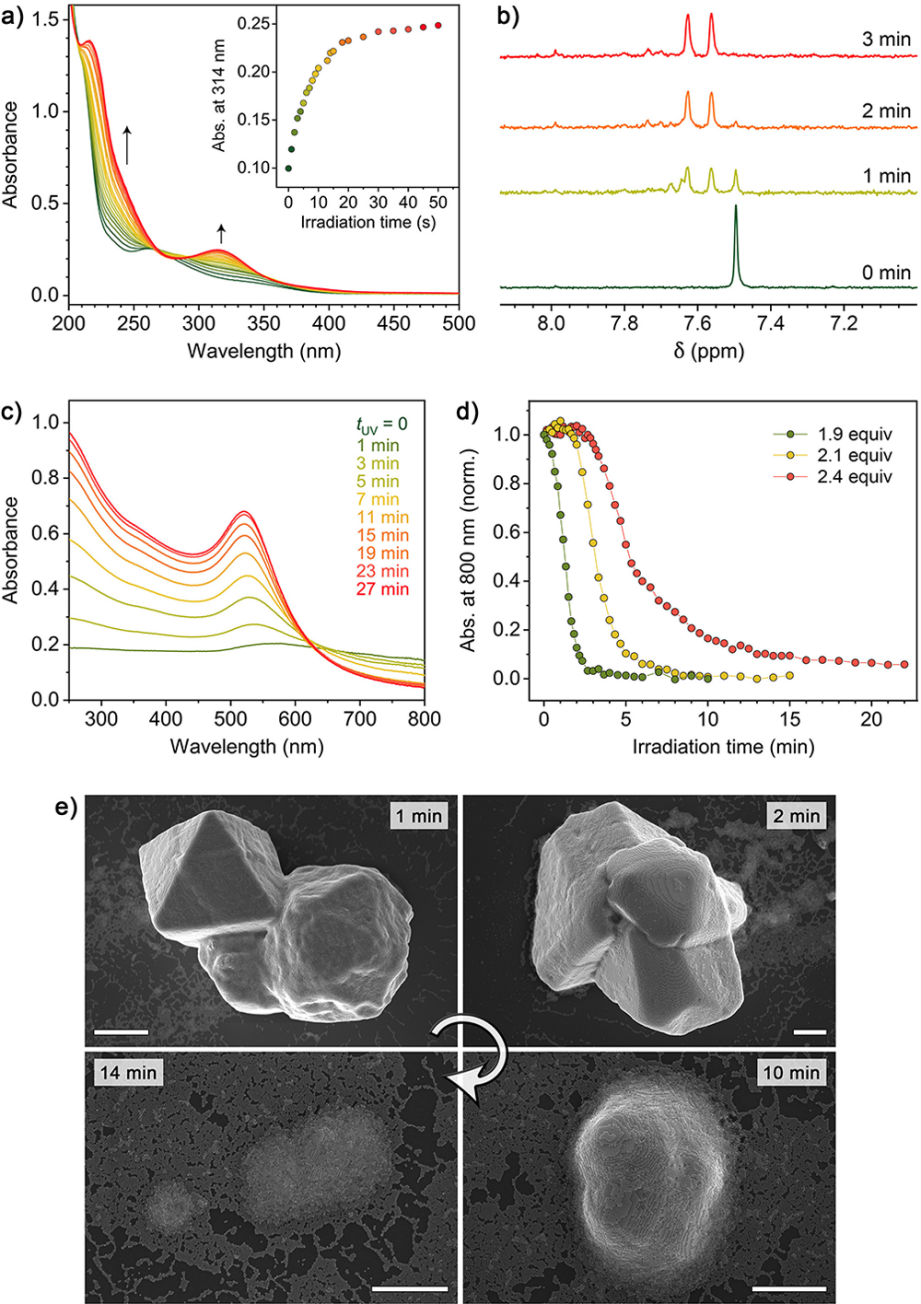
Light-induced disassembly of Au·TMA/NBTS aggregates. (a) Evolution of the UV/vis spectra of an aqueous solution of NBTS (trisodium salt) during UV (365 nm) irradiation (for the reaction equation, see Figure 1c). (b) Partial ^1^H NMR spectra of NBTS in CD_3_OD before (bottom) and after different times of UV irradiation. (c) Evolution of UV/vis spectra of an aqueous suspension of Au·TMA/NBTS aggregates (made from 5.3 nm NPs) during UV irradiation for up to 27 min. It is worth pointing out that we found no significant differences between the disassembly kinetics of amorphous and crystalline Au·TMA/NBTS aggregates (Figure S9). (d) Disassembly profiles of Au·TMA/NBTS aggregates in the presence of increasing amounts of NBTS. (e) Representative SEM images of the crystalline Au·TMA/NBTS aggregates after UV exposure for increasing periods (scale bars = 500 nm).

The above experiments demonstrate that the NBTS trianion behaves as an “anionic glue” for cationic gold NPs, similar to trianions reported previously.^63^ To determine whether NBTS can also act as a *photocleavable* anionic glue, we first studied its stability under UV (365 nm) light. Figure 4a shows a series of UV/vis spectra of an aqueous solution of NBTS (trisodium salt) under UV irradiation (we worked with low-intensity (∼1 mW·cm^–2^) light-emitting diodes). We found that NBTS’s characteristic absorption band at ∼270 nm gradually disappeared and that a more intense, red-shifted peak centered at ∼315 nm grew (along with additional absorption features below 250 nm). No further changes in the spectra were observed after 1 min of irradiation, indicating that the reaction had reached completion. We also followed the reaction by NMR spectroscopy. The partial ^1^H NMR spectra focusing on the high-ppm region (i.e., aromatic protons; Figure 4b) show a gradual appearance of two singlets (at 7.56 and 7.63 ppm) at the expense of one (at 7.50 ppm). The light-induced transformation of NBTS into NBDS (Figure 1c) is accompanied by desymmetrization of the molecule, consistent with the NMR spectra before and after the reaction.

The green trace in Figure 4c (*t*_UV_ = 0) shows a featureless UV/vis spectrum of Au·TMA/NBTS aggregates suspended in water. Exposing this sample to UV light for ∼20 min resulted in a spectrum practically identical to that of an aqueous suspension of Au·TMA before NBTS was added (Figure 2a), suggesting that the assembly–disassembly cycle did not affect the NP integrity. Indeed, analysis of Au·TMA by TEM before the addition of NBTS and after UV irradiation revealed that the NPs had the same size and size dispersity (Figure S8). Notably, the disassembly of NP aggregates took significantly longer than the photocleavage of free NBTS (∼20 min vs <1 min; Figure 4c and a, respectively). This significant difference suggests that NBTS complexed with NPs is resistant (or less prone) to photodecomposition due to the gold NPs’ high absorptivity in the UV region. Consequently, the disassembly of NP aggregates is likely driven by the photocleavage of free NBTS and gradually shifting the Au·TMA/NBTS ⇌ (Au·TMA)^3*n*+^ + *n* NBTS^3–^ equilibrium to the right.

The preferential decomposition of unbound NBTS led us to speculate that it might be possible to tune the onset of NP aggregates’ disassembly by controlling the amount of NBTS present in the system. To test this hypothesis, we titrated three identical samples of Au·TMA with NBTS until 1.9, 2.1, and 2.4 equiv of NBTS were added. Indeed, the “lifetime” of the resulting NP aggregates was proportional to the amount of NBTS added. Interestingly, we also found that the delayed disassembly was accompanied by slower disassembly kinetics (manifested by the different slopes of the disassembly profiles; see Figure 4d). This result can be attributed to the aging of the Au·TMA/NBTS aggregates. We note that NBTS is a highly flexible molecule; over time, it can adopt a conformation optimal for maximizing the electrostatic interactions with NPs’ charged TMA headgroups (in fact, we have previously postulated^63,72^ that these “ionic glues” have a dynamic character in that they constantly bind to and unbind from the NPs). This hypothesis is further supported by our experiments with the significantly more rigid “inorganic PAG”, as discussed in the next section.

We performed a series of control experiments to verify that the disassembly of NP aggregates results from the UV-induced decomposition of NBTS trianion into NBDS and succinate dianions. First, we found that the Au·TMA/NBTS aggregates did not decompose in the dark or upon irradiation with various wavelengths of visible light (thus eliminating the effect of plasmonic heating; Figure S10). In addition, (i) no visual changes were observed after several weeks in the dark or under ambient conditions (fluorescent laboratory light), and (ii) no noticeable temperature increase was found upon exposing free Au·TMA to UV light (Figure S11). Second, Au·TMA did not aggregate when titrated with a partially protonated NBTS (a mixture of monoanion and dianion; the pH was adjusted to 5; Figure S12). Similarly, no aggregation was observed upon treating Au·TMA with NBTS pre-exposed to UV light (i.e., a mixture of NBDS and succinate) (Figure S13). Finally, aggregates in which the same 5.3 nm Au·TMA NPs were “glued” through a non-photocleavable trianion (here, we used citrate) did not show any appreciable response to UV light under the same irradiation conditions (Figure S14).

The light-induced disassembly of Au·TMA/NBTS aggregates could also be followed by SEM (Figure 4e). In these experiments, we exposed crystalline aggregates to UV light for increasing periods, after which we drop-casted the samples onto a silicon wafer and rapidly evaporated the solvent. A representative series of SEM images in Figure 4e shows that the crystals disassembled isotropically and became gradually less faceted, before they gave rise to a solution of free NPs.

The resulting NPs could be reassembled into photoresponsive aggregates by adding a fresh aliquot of NBTS; moreover, the subsequent addition and spontaneous evaporation of (NH_4_)_2_CO_3_ resulted in aggregates that were both light-sensitive and crystalline (Figure S15). However, the degree of crystallinity was noticeably lower than in the first cycle, which can be explained by the accumulation of NBDS and succinate (which interfere with the NP crystallization process). In contrast, the system’s photoresponsive character persisted for many assembly–disassembly cycles (*vide infra*; Figure S19).

### An Inorganic Photocleavable Anionic Glue

To expand the concept of PAG, we worked with potassium trioxalatocobaltate (TOC). The light-induced photodecomposition of TOC has been known for more than a century,^73^ and its mechanism has been investigated in detail.^74,75^ Upon exposure to light, TOC undergoes disproportionation, whereby Co(III) is reduced to Co(II), and a fraction of oxalate is oxidized to CO_2_ (Figure 1d). Co(II) exists preferentially as a dioxalato–diaqua complex (DOC in Figure 1d), which has a net charge of −2 (Figure 1d). Therefore, we hypothesized that TOC should similarly act as a photocleavable anionic glue.

**Figure 5:**
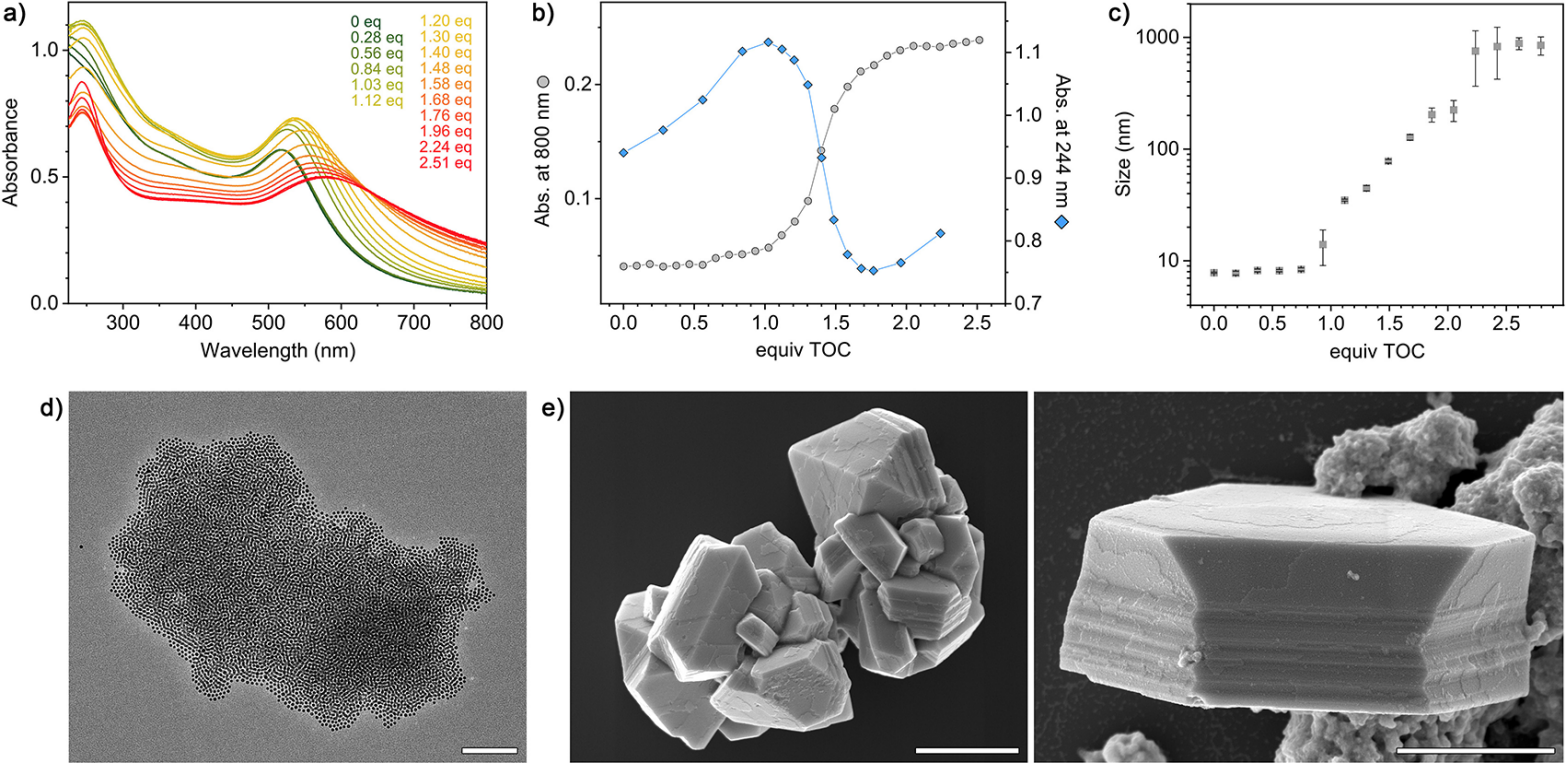
Self-assembly of positively charged nanoparticles (5.3 nm Au·TMA) mediated by TOC. (a) A series of UV/vis absorption spectra recorded during the gradual addition of TOC to a solution of 5.3 nm Au·TMA. The prominent absorption peak at ∼245 nm originates from TOC. (b) Changes in the absorbance at 800 nm (gray markers) and 244 nm (blue markers) during the titration of 5.3 nm Au·TMA with TOC (“equiv TOC” denotes the molar ratio of negative charges (added as TOC) to positive charges (the total number of NP-adsorbed TMA ligands in the titrated solution). (c) Changes in the hydrodynamic diameter of 5.3 nm Au·TMA NPs or their aggregates during the titration of the NPs with TOC. (d) A representative TEM image of an amorphous Au·TMA/TOC aggregate (NP size = 5.3 nm; scale bar 100 nm). (e) Representative SEM images of colloidal crystals coassembled from 5.3 nm Au·TMA and TOC (scale bars = 1 μm).

To this end, we first studied the titration of 5.3 nm Au·TMA with TOC. The series of UV/vis spectra in Figure 5a are reminiscent of those in Figure 2a; however, TOC’s intense absorption peak at ∼244 nm allowed us to study the assembly process in more detail. In Figure 5b, we plotted the change in absorbance at 800 nm (*A*_800_; proportional to the degree of NP aggregation) and at 244 nm (*A*_244_; proportional to the concentration of the soluble fraction of TOC) as a function of the amount of TOC titrant added. When >1.0 equiv of the titrant was added (defined as the molar ratio of negative charges on TOC to the positive charges on NP-immobilized TMA ligands), absorbance at 800 nm increased sharply as NP aggregation commenced. At the same time, *A*_244_ dropped abruptly; although the addition of TOC was continued (i.e., a steady increase of *A*_244_), we note that the absorptivity of the NP aggregates in this region is much lower than that of free gold NPs. The subsequent addition of TOC resulted in a further increase of *A*_244_; the newly added TOC carried an excess of negative charge (with respect to the positive charge on the NPs) and thus did not interact with the electroneutral Au·TMA/TOC aggregates. The assembly process could also be followed by dynamic light scattering (DLS); as shown in Figure 5c, the hydrodynamic diameter started to increase after 1.0 equiv of TOC was added.

Figure 5d shows a TEM image of a typical Au·TMA/TOC aggregate obtained by mixing the two species; as expected, the NPs within these aggregates are disordered. However, we succeeded in transforming these amorphous aggregates into highly crystalline ones using the strategy outlined in Figure 3a. Similar to the Au·TMA/NBTS system, the resulting crystals exhibit morphologies typical of the fcc phase (Figure 5e; for additional examples, see Figure S16). The successful formation of well-defined crystals is an interesting result, given that the process involves a saturated (∼10.5 M) solution of (NH_4_)_2_CO_3_. We experimentally determined that the coassembly of Au·TMA and TOC in a solution of evaporating ammonium carbonate is initiated when the concentration of (NH_4_)_2_CO_3_ is still high: 125 ± 5 mM, which corresponds to the CO_3_^2–^/C_2_O_4_^2–^ molar ratio of ∼10^4^. However, the high stability of TOC^76^ (log β_3_ ≈ 31) means that CO_3_^2–^ is outcompeted in terms of binding to Co(III), even in the presence of a massive excess of carbonate anions.

**Figure 6:**
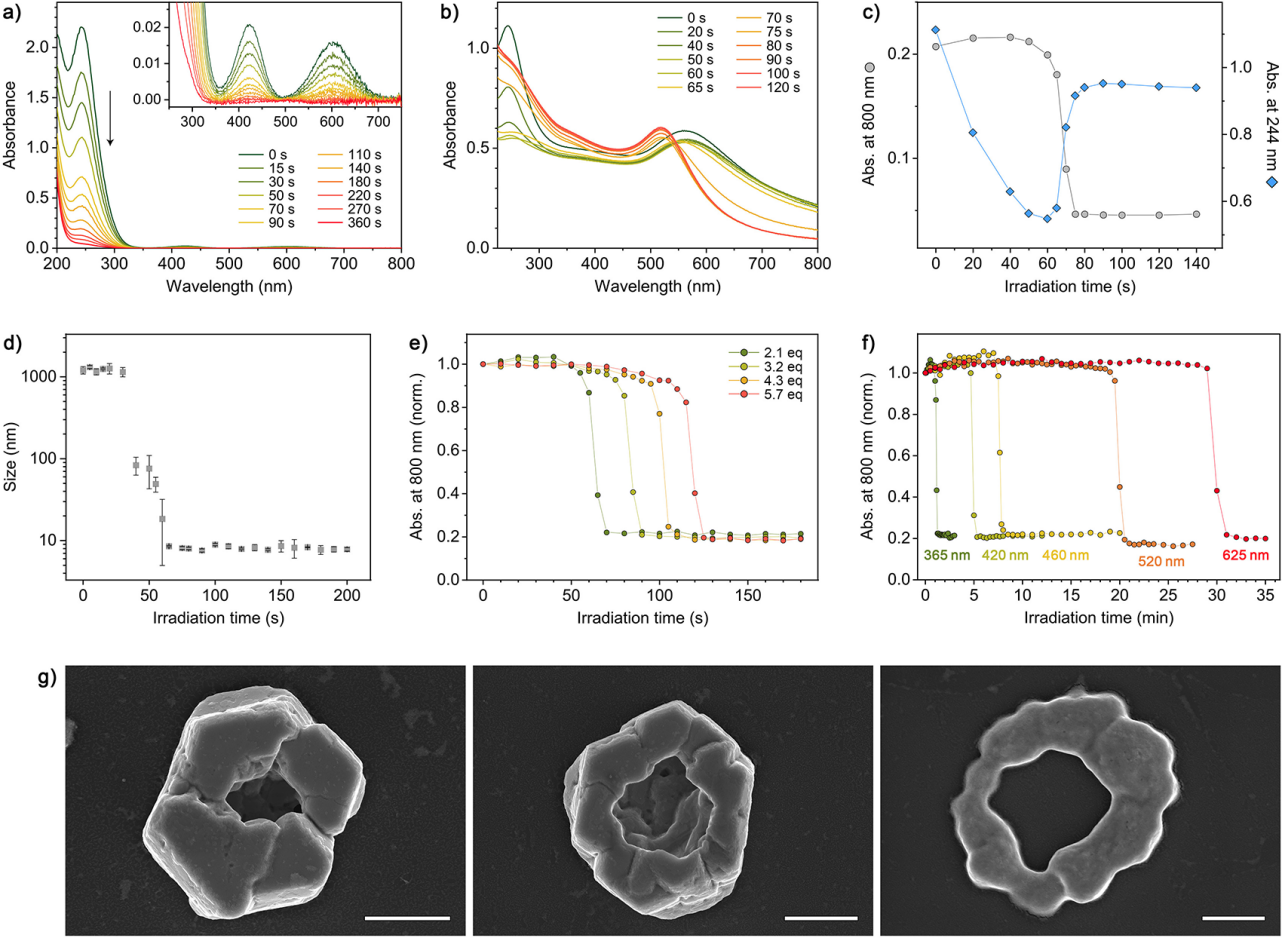
Light-induced disassembly of Au·TMA/TOC aggregates. (a) A series of UV/vis spectra accompanying the UV (365 nm) irradiation of an aqueous solution of TOC (tripotassium salt) for up to 6 min (for the reaction equation, see Figure 1d). (b) A series of UV/vis spectra accompanying the UV irradiation of Au·TMA/TOC aggregates (obtained from 5.3 nm NPs and ∼2 equiv of TOC). (c) Changes in the absorbance at 800 and 244 nm (gray and blue markers, respectively) during the UV irradiation of Au·TMA/TOC aggregates. (d) The hydrodynamic diameter of Au·TMA/TOC aggregates as a function of the UV irradiation time. (e) Tuning the onset of disassembly of Au·TMA/TOC aggregates by the excess of TOC under the same irradiation conditions (the number of TOC equivalents denotes the molar ratio of negative charges (three times the amount of TOC) with respect to the number of NP-adsorbed TMA ligands). (f) Tuning the onset of Au·TMA/TOC aggregate disassembly by the wavelength of incident light (in all cases, ∼2 equiv TOC was used). For full-range spectra, see Figure S18. (g) SEM images of Au·TMA/TOC aggregates (obtained from 5.3 nm NPs) following UV light irradiation before the aggregates disassembled completely (scale bars = 1 μm).

Upon irradiation with UV light, TOC’s intense absorption peak at ∼244 nm is quenched (Figure 6a) − a signature of the photochemical decomposition to DOC (Figure 1d). To follow the light-induced disassembly of Au·TMA/TOC aggregates, we titrated Au·TMA with TOC until an excess (∼2 equiv) of the titrant was added and then exposed the resulting solution to UV light (Figure 6b). During the initial 60 s of irradiation, *A*_244_ steadily decreased, but the aggregates remained intact (see the blue and gray markers, respectively, in Figure 6c). At ∼60 s, *A*_800_ dropped precipitously, indicating the disassembly of NP aggregates into free Au·TMA (which absorb much stronger in the UV region, hence the sharp increase in *A*_244_). The disassembly process could also be monitored by DLS (Figure 6d).

To verify whether the lifetimes of Au·TMA/TOC aggregates can be tuned by the amount of extra TOC (analogously to the NBTS system), we titrated Au·TMA with TOC until increasing excesses of the titrant had been added and then subjected the resulting suspensions to UV light. As expected, the onset of disassembly depended on the amount of TOC; for example, with 2.1 equiv and 5.7 equiv of TOC, disassembly commenced after ∼50 s and ∼110 s, respectively (Figure 6e). The subsequent addition of a fresh aliquot of TOC to the resulting free Au·TMA induced their reassembly, and the light-induced disassembly–reassembly process could be repeated for at least several cycles. Interestingly, we observed no noticeable fatigue after five such cycles; see Figure S19.

The use of TOC offers another way to control aggregate lifetimes. In contrast to NBTS, which can only be cleaved with UV light, TOC has additional absorption bands spanning the entire visible region (Figure 6a, inset), which led us to hypothesize that Au·TMA/TOC aggregates can be disassembled with various wavelengths of light (see Figure S17). Indeed, Au·TMA/TOC aggregates disassembled into free NPs upon exposure to all tested colors of light: 365 nm (UV), 420 and 460 nm (blue), 520 nm (green), and 625 nm (red). However, the onset of disassembly was heavily dependent on the LED wavelength; for example, red light induced disassembly after ∼30 min, but less than 1 min of UV irradiation was sufficient to complete the disassembly (Figure 6f) (the intensities of all the LEDs were similar, at ∼1 mW·cm^–2^). These results can be explained by the strong dependence of the quantum yield of TOC decomposition on the incident photon wavelength.

Interestingly, the slope in the Au·TMA/TOC disassembly profiles showed no noticeable dependence on the amount of TOC or aging time (Figure 6e,f). In all cases, the slopes were steep; that is, disassembly was initiated at a specific time and was completed soon afterward − a sought-after feature for controlled-release applications. This behavior differs from that of aged Au·TMA/NBTS aggregates, which took significantly more time to disassemble completely (Figure 4d). To explain these results, we note that the TOC trianion is substantially more rigid than the flexible NBTS. In addition, although both PAGs carry three negative charges, the charges in NBTS are localized on the terminal carboxylate groups, facilitating Coulombic interactions with NPs’ positively charged TMA groups (as opposed to solely neutralizing NPs’ positive charge). Consequently, NBTS behaves as a more “persistent” ionic glue; in contrast, TOC is more likely than NBTS to unbind from NPs rapidly. The more TOC is unbound and decomposed to DOC, the lower the TOC/TMA ratio; the electroneutrality condition is no longer obeyed, which facilitates the disassembly of the Au·TMA/TOC aggregates even further.

We conclude this section by reporting an unexpected observation. We attempted to follow the UV-induced disassembly of crystalline Au·TMA/TOC assemblies by SEM, focusing on the relatively short period between the initiation and completion of disassembly. The samples collected during this period contained a significant fraction of crystals with large cavities on their flat faces (Figure 6g). No such structures were ever found in experiments with the Au·TMA/NBTS crystals, which disassemble preferentially at the most exposed locations, such as the edges and corners (Figure 4e). Based on our observations, we conclude that the disassembly of hexagonal-crystal Au·TMA/TOC assemblies begins near the center of the large-face centers. We hypothesize that cavity formation at these sites is related to the fast disassembly kinetics of Au·TMA/TOC aggregates. The desorption of the outer layer of TOC trianions creates a transient excess of positive charge (due to TMA); charge accumulation is least favorable at the centers of flat surfaces, expelling the NPs (Coulombic repulsion) from these locations. This speculation is in agreement with the dynamic^77^ nature of the TOC ionic “glue” (and the sharp disassembly profiles of Au·TMA/TOC aggregates).

### A Combination of NBTS and TOC Enables Sequential Disassembly of NP Aggregates

**Figure 7:**
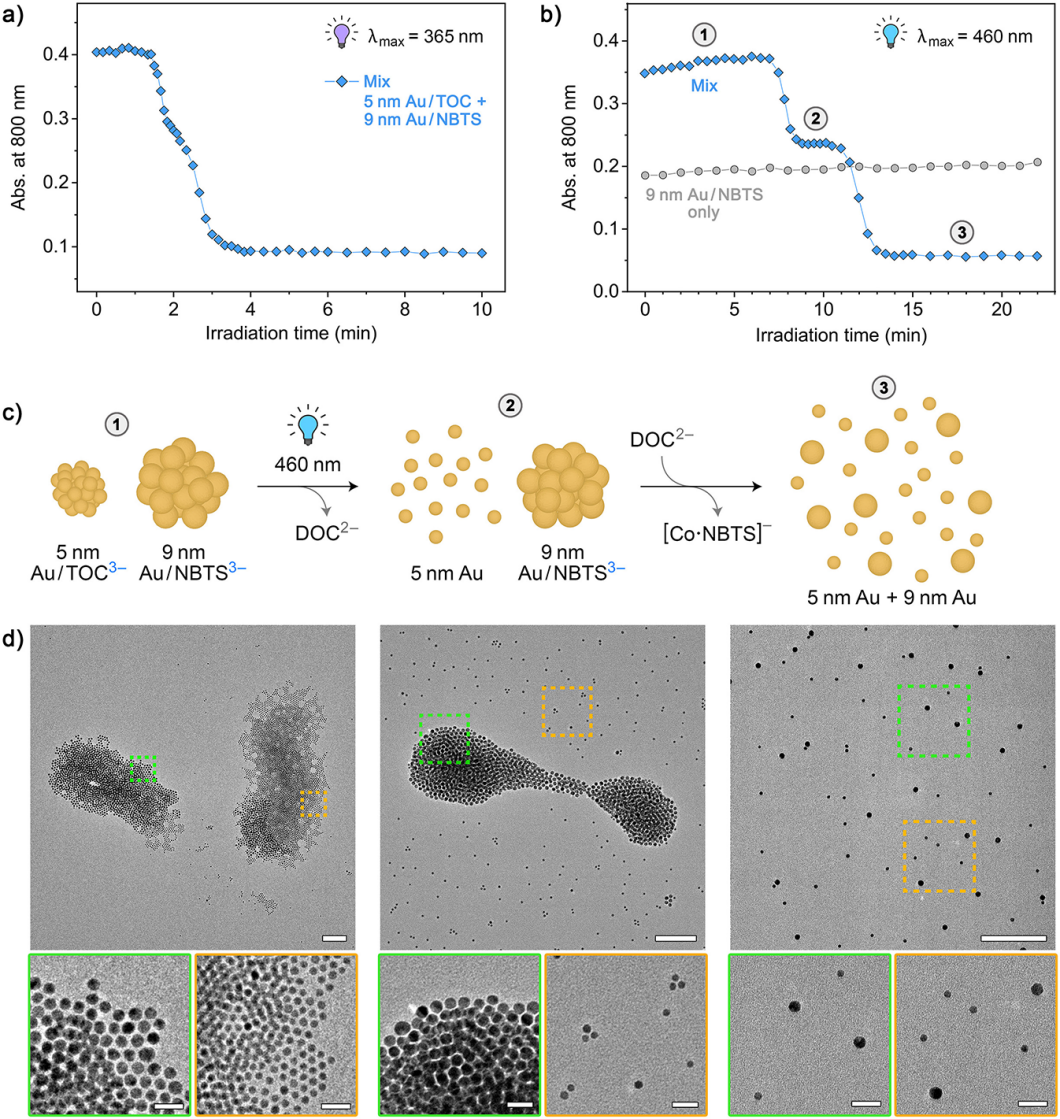
Light-induced sequential disassembly of nanoparticle aggregates. (a) The disassembly profile of a mixture of two types of aggregates (5.3 nm Au·TMA/TOC + 9.5 nm Au·TMA/NBTS) under UV light. For full-range spectra, see Figure S21. (b) The disassembly profile under blue light (460 nm) for a mixture of two types of aggregates (5.3 nm Au·TMA/TOC + 9.5 nm Au·TMA/NBTS, “mix”) (blue markers). Gray markers: the behavior of the same Au·TMA/NBTS aggregates in the absence of Au·TMA/TOC. For full-range spectra, see Figure S22. (c) Schematic representation of the mechanism underlying the sequential disassembly of a mixture of Au·TMA/TOC and Au·TMA/NBTS under blue light. (d) Representative TEM images of the 5.3 nm Au·TMA/TOC + 9.5 nm Au·TMA/NBTS mixture before (left; stage 1 in panel b) and after exposure to blue light for 10 min (center; stage 2 in b) and 20 min (right; stage 3 in b). The bottom-row images are the magnified views of the regions denoted by dashed lines (scale bars = 100 nm in the main images and 20 nm in the magnified images).

Finally, we considered combining both types of PAG—organic and inorganic—in a single system. Although it is not possible to address NBTS and TOC in an orthogonal fashion^78^ (i.e., UV light, required to cleave NBTS, also decomposes TOC), we hypothesized that exposing a mixture of the two aggregates—Au/NBTS + Au/TOC—to visible light might selectively liberate NPs from the latter ones. To conveniently visualize this process, we worked with two monodisperse batches of Au NPs, 5.3 nm Au·TMA and 9.5 nm Au·TMA. First, we independently titrated the smaller NPs with TOC and the larger ones with NBTS until ∼2.1 equiv of the titrant was added in both cases. Then, the two types of aggregates were mixed (in a 1:1 ratio with respect to the total number of Au atoms, such that both the 5.3 and 9.5 nm NPs contribute roughly equally to the sample’s absorbance) and subjected to irradiation. TEM imaging before irradiation confirmed that the small and the large NPs were localized in separate aggregates (Figure 7d, left), although some mixing was observed (Figure S20).

Figure 7a shows the change in *A*_800_ under UV light. After the initial lag period (during which the unbound NBTS and TOC were decomposed), *A*_800_ dropped sharply. It is interesting to note that the curve has two slopes, which correspond to the disassembly of the two types of aggregates. Then, the same Au/NBTS + Au/TOC aggregate mixture was exposed to 460 nm light (Figure 7b). After ∼7 min of irradiation, *A*_800_ decreased by approximately half the amount it had dropped under UV light (stage **2** in Figure 7b and c), indicating that roughly half of the total amount of Au was liberated from the aggregates as free NPs, as expected from the selective photodecomposition of TOC and the release of the smaller NPs. Indeed, inspection of this sample by TEM revealed that only aggregates of 9.5 nm Au·TMA (held together by NBTS) remained (Figure 7d, center). Surprisingly, however, continued irradiation resulted in an additional drop in *A*_800_, down to the value expected from a solution containing no aggregates, as confirmed by TEM (Figure 7d, right). The disassembly of Au/NBTS was not due to the photochemical cleavage of NBTS, as confirmed by a control experiment, in which Au/NBTS aggregates without Au/TOC were exposed to 460 nm light (no changes were found; gray markers in Figure 7b). Instead, we note that the affinity of oxalate to Co^2+^ is much lower^79,80^ than to Co^3+^ (log β_3_ ≈ 31 for TOC^76^ vs log β_2_ ≈ 6.4 for DOC^81^). At the same time, we wish to point out that in addition to interacting with Au·TMA electrostatically, NBTS can complex Co^2+^ ions. In fact, a related tricarboxylate, nitrilotriacetate N(CH_2_COO^–^)_3_, binds to Co^2+^ with log β_2_ ≈ 13.9.^82^ Therefore, we propose that NBTS outcompetes oxalate in terms of binding to Co^2+^, thus losing its ionic-glue character (Figure 7c). In other words, Co^2+^ outcompetes Au·TMA in terms of interaction strength with NBTS, effectively extracting it from the Au/NBTS aggregates and thus inducing their disassembly.^83^

We conducted the following control experiments to verify the above mechanism. First, we treated 9.5 nm Au/NBTS aggregates with an aqueous solution of TOC pre-exposed to UV light and found that they slowly disassembled (Figure S24a). In this experiment, the NP aggregates were not exposed to light; therefore, their disassembly must have been induced by a product of TOC decomposition (i.e., DOC). Second, we similarly decomposed TOC into DOC and mixed the resulting solution with 5.3 nm Au·TMA (at the same ratio as in Figure 7b). As expected, the NPs did not assemble. Then, we added this solution to a suspension of 9.5 nm Au/NBTS, and we found that these aggregates disassembled rapidly (within 30 s; Figure S24b). This result can be explained by the insufficient availability of ionic glue (here, NBTS) to sustain Au·TMA in the assembled state; in fact, by treating the Au/NBTS assemblies with free Au·TMA, we reduced the fraction of PAG (expressed as “equiv NBTS” in Figure 2b) from ∼2.1 equiv to ∼0.5 equiv − a regime in which the NPs remain disassembled (see Figure 2b). Taken together, these two experiments demonstrated that the second disassembly step (Figure 7b) is driven by a combination of (i) the sequestration of the NBTS ionic glue by Co^2+^ and (ii) departure from the electroneutrality condition. Finally, we note that according to the proposed mechanism, irradiation is not required for the second disassembly step. Therefore, in the third control experiment, we repeated the experiment shown in Figure 7b, but turned off the light as soon as the first plateau (stage 2) was reached. Indeed, we found that the aggregates of the second type also disassembled in the dark, after the initial exposure to visible light (Figure S24c).

## Conclusions

In summary, we described the concept of photocleavable ionic glue, i.e., light-sensitive trianions capable of inducing the aggregation of positively charged nanoparticles prior to—but not after—light irradiation. When titrated with these trianions, cationic NPs aggregated readily near the electroneutrality point. The resulting aggregates were amorphous, but could be converted into highly crystalline ones by rapidly increasing and then slowly decreasing the solution’s ionic strength. NPs having different sizes (5.3 and 9.5 nm) and PAGs as structurally diverse as a rigid cobaltate complex and a flexible nitrobenzyl derivative behaved similarly. Upon exposure to light, both PAGs are converted into dianionic products, incapable of maintaining the attractive interparticle interactions, resulting in disassembly. Because the degree of NP aggregation shows a highly nonlinear dependence on the PAG concentration, the NP aggregates disassembled abruptly once a critical amount of PAG had been decomposed. Despite these analogies, the two PAGs also showed some differences. First, whereas the nitrobenzyl PAG is only sensitive to UV light, the cobalt complex absorbs and can be decomposed using all the wavelengths in the near-UV and the visible region (with the onset of disassembly strongly dependent on and tunable by the color of the incident light). Second, the inorganic PAG is considerably more rigid than the organic PAG, resulting in less pronounced tails in the disassembly profiles under the same UV irradiation conditions. Finally, we showed that by combining the two PAGs in a single system, it is possible to engineer complex disassembly—and therefore release—profiles, of potential relevance for controlled-delivery applications. Future work will focus on coupling the light-induced disassembly of the NP/PAG aggregates to various biological molecules and processes.

## Supporting Information Available

General experimental remarks, synthetic procedures and characterization, SEM and TEM images of NPs and NP assemblies, control experiments (PDF)

## ABBREVIATIONS

DLS: dynamic light scattering
DOC: dioxalatocobaltate(II)
NBDS: nitrosobenzyldisuccinate
NBTS: nitrobenzyltrisuccinate
NP: nanoparticle
TMA: (11-mercaptoundecyl)-*N*,*N*,*N*-trimethylammonium
TOC: trioxalatocobaltate(III)
PAG: photocleavable anionic glue
SEM: scanning electron microscopy
TEM: transmission electron microscopy
SPR: surface plasmon resonance
UV: ultraviolet
vis: visible
