# Supplementary material for: Photocleavable Anionic Glues for Light-Responsive Nanoparticle Aggregates
Source: J Am Chem Soc. 2023 Feb 9;145(7):4098–108. doi: 10.1021/jacs.2c11973 (PMC9951211; doi:10.1021/jacs.2c11973)
Supplement: Supplementary file 1 — ja2c11973_si_001.pdf [file ja2c11973_si_001.pdf]

Photocleavable Anionic Glues for Light-Responsive Nanoparticle Aggregates

Jinhua Wang, Tzuf Shay Peled, and Rafal Klajn*

Department of Organic Chemistry, Weizmann Institute of Science, Rehovot 76100, Israel

*e-mail: rafal.klajn@weizmann.ac.il

Table of contents:

1. General remarks 2

2. Synthesis of photocleavable anionic glues (PAGs).. 2

  2.1. NBTS – the organic PAG.. 2

  2.2. TOC – the inorganic PAG.. 3

3. Synthesis of and functionalization of Au·TMA 3

4. NBTS-mediated assembly and light-induced disassembly of Au NPs 7

5. TOC-mediated assembly and light-induced disassembly of Au NPs 14

6. Experiments with mixtures of the two PAGs.. 17

7. Supporting references.. 20

1. General remarks

All commercially available chemicals were used as received. NMR spectra were recorded on a Bruker Avance III 300 MHz spectrometer. Chemical shifts (δ) are given in ppm relative to residual protio solvent resonances (3.31 ppm for CD_3OD and 7.26 ppm for $CDCl_3$). UV/vis absorption spectra were recorded on an Agilent Cary 60 spectrophotometer. For the photoirradiation experiments, we used Prizmatix mic-LED 365 nm, 420 nm, 460 nm, 520, and 625 light-emitting diodes (LEDs). The electrospray ionization mass spectrometry (ESI-MS) experiments were carried out on a Bruker Daltonics Esquire 300 Plus ESI mass spectrometer using spectroscopic-grade methanol. Thermogravimetric analysis (TGA) was performed on a Thermal Analysis SDT Q600 system, with a heating rate of 10 ºC/min from room temperature to 800 ºC and an air flow of 100 mL/min. Inductively coupled plasma mass spectrometry analysis was performed on an Agilent 7700s ICP-MS system. Dynamic light scattering (DLS) and ζ-potential measurements were carried out on a Malvern Zetasizer Nano ZS instrument. Transmission electron microscopy (TEM) was carried on a Thermo Fisher Talos F200x microscope and scanning electron microscopy (SEM) was carried out on a Zeiss Sigma 500 and a Zeiss Ultra 55 microscope.

2. Synthesis of photocleavable anionic glues (PAGs)

2.1. NBTS – the organic PAG

NBTS was synthesized according to the scheme below, starting from the commercially available 1,3,5-tris(bromomethyl)benzene **1**. Compounds **2** and **3** were synthesized by modifying the procedures described in refs. 1 and 2, respectively.

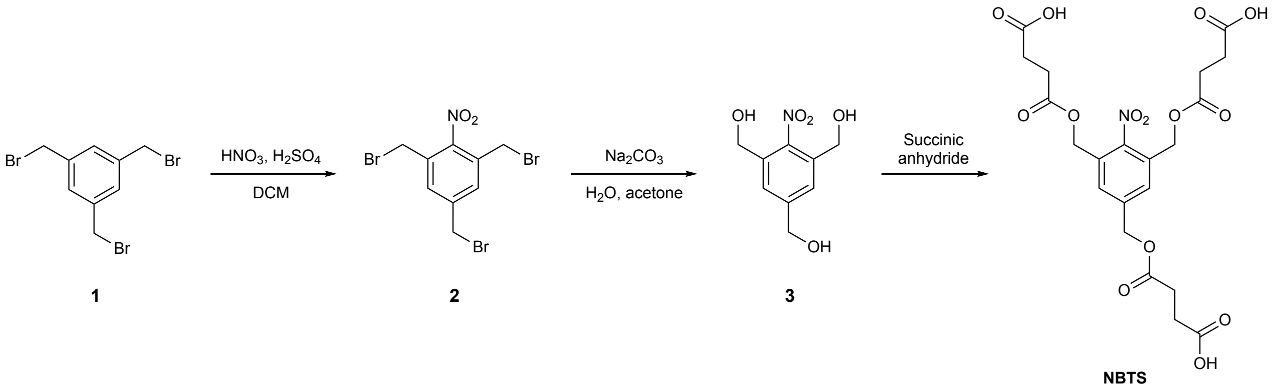

2: Compound **1** (1.07 g, 3.0 mmol) was dissolved in 50 mL of dichloromethane and the solution was cooled to ~5 ºC with an ice bath. Concentrated sulfuric acid (1.8 mL) was added slowly, followed by concentrated nitric acid (1.8 mL, dropwise addition), and the resulting mixture was stirred at room temperature for ~2 h. Then, the reaction was quenched with 30 mL water. The organic phase was collected and washed with water, then with a diluted solution of Na_2CO_3 (2 × 30 mL), dried over $MgSO_4$, and the solvent was removed *in vacuo*. The crude residue was purified by silica gel column chromatography using dichloromethane/hexane (1:4, *v*/*v*) as the eluent. The solvent was evaporated *in vacuo* and the product was collected as a light-yellow solid (yield: 1.14 g, 94%). ^{1}H NMR ($CDCl_3$, 300 MHz, 298 K): δ (ppm) = 7.52 (s, 2 H), 4.48 (s, 4 H), 4.45 (s, 2 H).

3: Compound **2** (1.24 g, 3.0 mmol) was dissolved in 30 mL of acetone and a solution of Na_2CO_3 (1.08 g) in 15 mL of water was added. The resulting mixture was stirred at 65 ºC for 3 days (until complete consumption of **2** was indicated by TLC analysis). Crude **3** was extracted with dichloromethane and the solvent was removed *in vacuo*. The

solid residue was purified by silica gel column chromatography using dichloromethane/methanol (5:1, *v*/*v*) as the eluent. The solvent was evaporated *in vacuo*, resulting in pure **3** (yield: 250 mg, 39 %). ^{1}H NMR (CD_3OD, 300 MHz, 298 K): δ (ppm) = 7.57 (s, 2 H), 4.69 (s, 4 H), 4.66 (s, 2 H).

NBTS: Compound **3** (60 mg, 0.28 mmol), succinic anhydride (170 mg, 1.69 mmol), 4-(dimethylamino)pyridine (DMAP, 6.0 mg, 0.042 mmol), and triethylamine (190 µL, 1.35 mmol) were placed in a dry flask containing a stirring bar. The solids were dissolved in acetonitrile (anhydrous, 5 mL), and the solution was refluxed overnight under a blanket of nitrogen. Then, the solvent was removed *in vacuo* and the crude solid was purified by C18 silica gel column chromatography using DCM/methanol (1:4 *v*/*v*) as the eluent. The fractions containing the product were collected, combined, and the solvent was removed *in vacuo*, resulting in a white solid (yield: 75 mg, 52 %). ^{1}H NMR (CD_3OD, 300 MHz, 298 K): δ (ppm) = 7.60 (s, 2 H), 5.23 (s, 6 H), 2.72–2.60 (m, 12 H); ^{13}C NMR (CD_3OD, 75 MHz, 298 K): δ (ppm) = 175.8, 173.5, 158.5, 141.8, 131.2, 129.8, 65.8, 63.2, 30.0, 29.8, 29.7, 29.6; ESI-MS calcd. for $C_{21}H_{22}NO_{14}$ $[M – H]^-$: 512.10, found: 512.26.

2.2. TOC – the inorganic PAG

Potassium trioxalatocobaltate (TOC) was synthesized according to a previously reported procedure.[3] Cobalt carbonate ($CoCO_3$, 0.2 mol; 23.8 g) was dissolved in a solution of oxalic acid ($H_2C_2O_4{\cdot}2H_2O$, 25.2 g) and potassium oxalate ($K_2C_2O_4{\cdot}H_2O$, 73.7 g) in 500 mL of hot water. The solution was cooled to 40 ºC and lead dioxide (PbO_2, 23.9 g) was added slowly with vigorous stirring, followed by glacial acetic acid (25 mL, dropwise). Stirring was continued for 1 h, during which the solution changed from red to dark-green. Undissolved PbO_2 was removed by filtration and ethanol (500 mL) was added to induce the precipitation of TOC as emerald-green needles. ICP-MS analysis: %Co calcd. for $K_3Co(C_2O_4)_3{\cdot}3H_2O$: 11.92%, found: 10.73±1.36%.

3. Synthesis of and functionalization of Au·TMA

Ligand synthesis: TMA-functionalized Au NPs (Au·TMA) were prepared by ligand exchange between oleylamine-capped Au NPs (Au·OLA) and (11-mercaptoundecyl)-*N,N,N*-trimethylammonium bromide (TMA; synthesized based on a previously reported procedure[4]), in the presence of a small amount of an electroneutral thiol (1-hexanethiol).

Nanoparticle synthesis: Au·OLA NPs were prepared by a seeded-growth approach reported previously.[5] Briefly, ~3 nm NPs ("seeds") were first prepared by reducing $HAuCl_4$ with borane–*tert*-butylamine complex in toluene in the presence of OLA at 25 ºC. Then, the seeds were purified, dissolved in a mixture of toluene and OLA in the presence of $HAuCl_4$, and heated overnight at 60 ºC (OLA acted as a weak reducing agent). The NP size was controlled by the $HAuCl_4$/seed (i.e., Au^{III}/Au^0) ratio. The NPs were purified by three cycles of precipitation with ethanol, filtration, and redispersion in toluene.

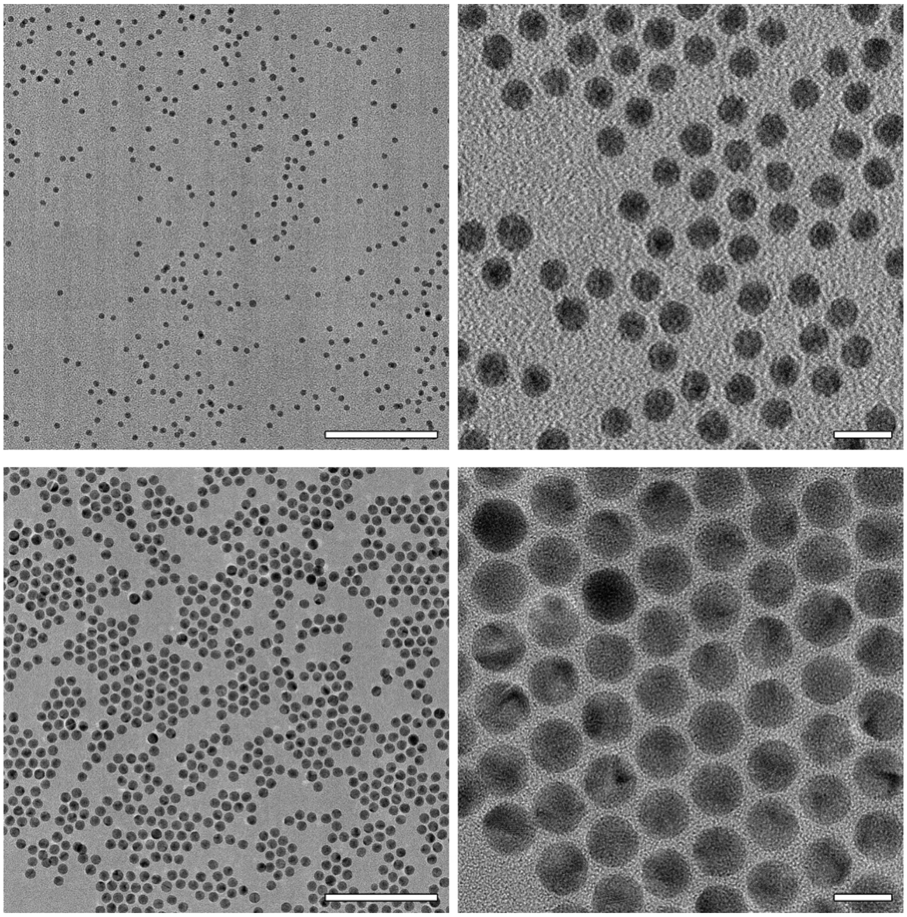

Figure S1. Representative TEM images of 5.32±0.39 nm Au·OLA (top) and 9.53±0.47 nm Au·OLA (bottom). The scalebars correspond to 100 nm (left panel) and 10 nm (right panel).

Nanoparticle functionalization: Au·TMA NPs were prepared by treating Au·OLA (in toluene) with mixture of 4.5 equiv of TMA and 0.5 equiv of HT (dissolved in an ethanol–toluene mixture; equiv with respect to the number of binding sites on the NPs, assuming that a single thiolate occupies[6] a surface area of 0.214 nm^2). After having been stirred vigorously for 2 h, the resulting precipitate was collected by centrifugation and redispersed in a small volume of methanol. The NPs were reprecipitated using ethyl acetate and collected by centrifugation. The redispersion–precipitation procedure was repeated twice. Finally, Au·TMA NPs were redispersed in a small volume of deionized water. The ζ-potential of the aqueous solutions of 5.32±0.44 nm Au·TMA and 8.93±0.46 nm Au·TMA was determined as +17.5±0.3 mV and +35.7±2.9 mV, respectively. We verified that the ligand exchange procedure did not affect the NPs' size and size dispersity.

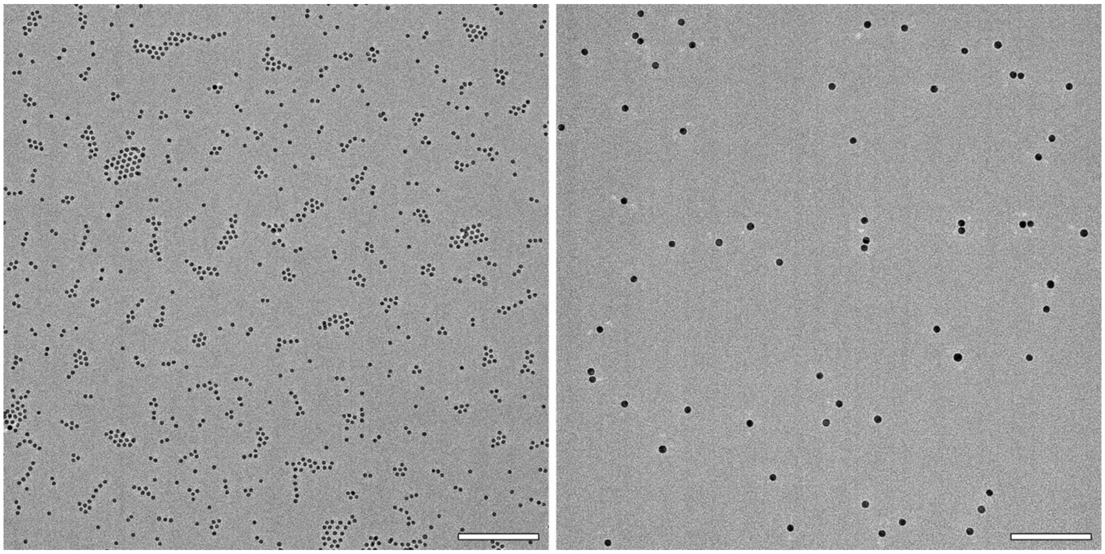

Figure S2. Representative TEM images of 5.32±0.44 nm Au NPs (left) and 8.93±0.46 nm Au NPs (right) co-functionalized with TMA and HT. The scalebars correspond to 100 nm.

Determination of the monolayer composition and ligand density on Au·TMA NPs: To determine the molar ratio of TMA to HT on the NPs, we adapted a previously reported procedure based on quantifying the ratio of two ligands following the oxidative dissolution of NPs with iodine.[7,8] 5.32±0.44 nm and 8.93±0.46 nm Au NPs (i.e., sizes similar to those in our self-assembly experiments) were synthesized and functionalized as described above (for the TEM images of functionalized NPs, see Figure S2), except that in the final step, they were not redispersed in water, but instead in DMSO-d_6. Then, excess (5 mg) of iodine was added and the solution was sonicated in a bath sonicator for 3 min, resulting in dissolution of the NPs and liberation of the NP-bound thiolates as the corresponding disulfides. The resulting solution was transferred into an NMR tube and an ^{1}H NMR spectrum was recorded (Figure S3). The molar ratio of TMA to HT was determined by integrating the signals at ~0.86 ppm (due to HT's methyl protons; 3 per HT) and ~3.03 ppm (due to TMA's methyl protons; 9 per TMA). The ratios amounted to 82:18 for 5.32 nm NPs and 86:14 for 8.93 nm NPs, respectively (note that these ratios are significantly lower than the 90:10 "feed" ratio, indicating the preferential adsorption of HT on the NPs). Next, thermogravimetric analysis (TGA) was performed to determine the combined fraction of organic ligands on the NPs. Prior to TGA, the NPs were dried under high vacuum (instead of dissolved in DMSO) to afford 4.610372 mg of dry 5.32 nm NPs and 6.641212 mg of dry 8.93 nm NPs. By combining the results of the TGA and NMR analysis, we conclude that the NPs used in our experiments are each decorated, on average, with ~370 TMA and ~81 HT ligands for the small NPs, and ~1046 TMA and ~170 HT ligands for the large NPs.

a)

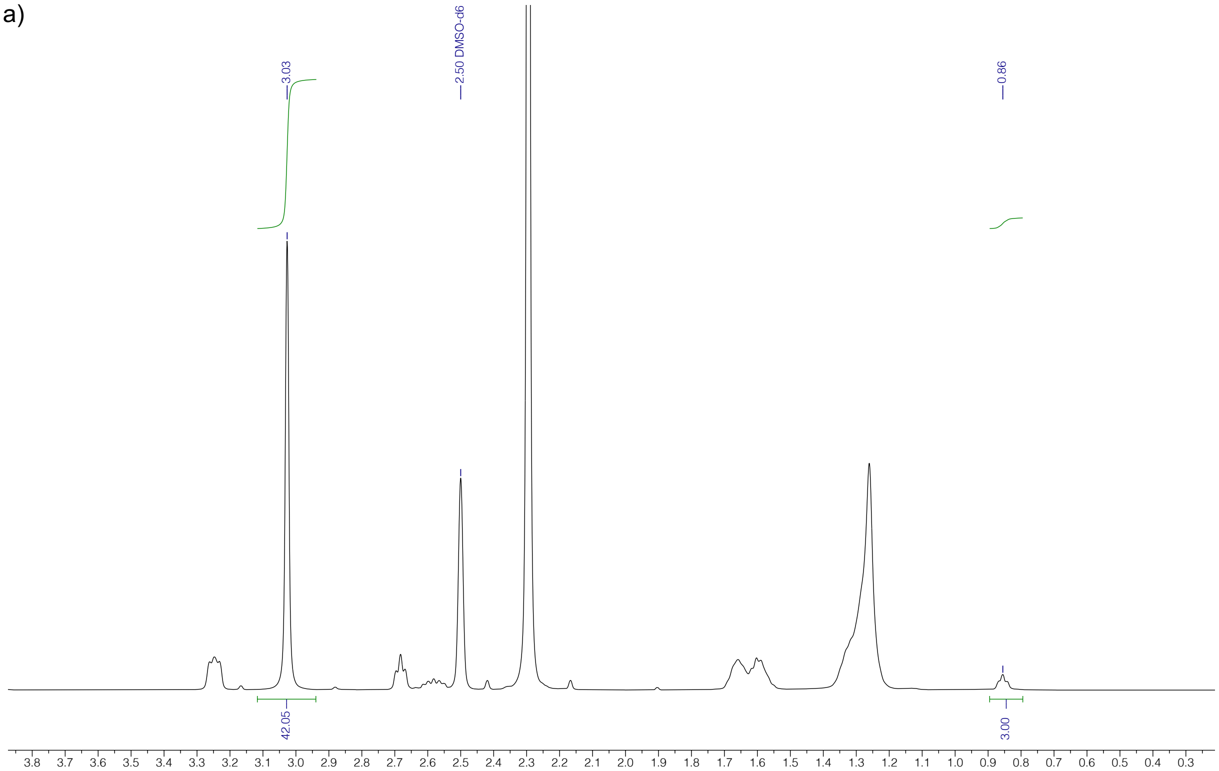

b)

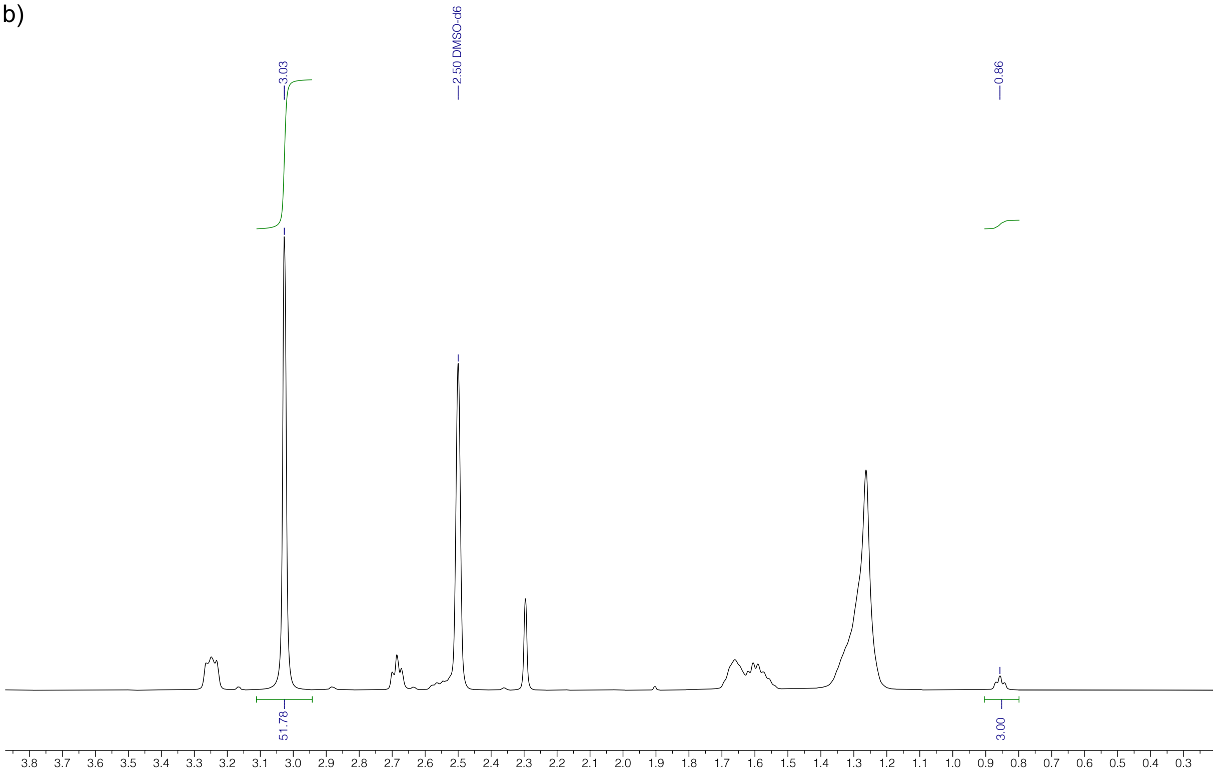

Figure S3. Partial ^{1}H NMR spectra of solutions obtained by iodine etching of (a) 5.32 nm and (b) 8.93 nm Au NPs functionalized with a 9:1 mixture of TMA and HT (500 MHz, DMSO-d_6). The signal at ~0.86 ppm originates from HT's methyl protons (t, 3H); the signal at ~3.03 ppm originates from TMA's methyl protons (s, 9H).

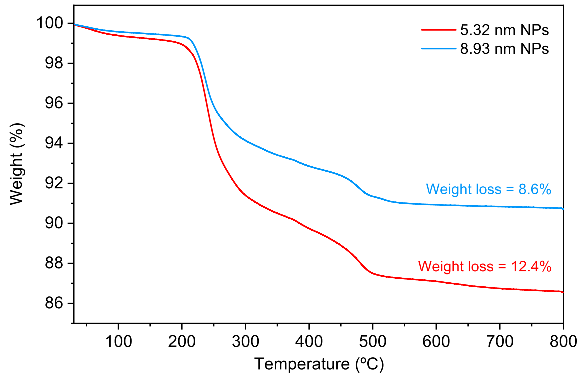

Figure S4. Thermogravimetric analysis (TGA) of Au NPs co-functionalized with TMA and HT. The TGA curves represent the percentage of the starting weight for the 5.32 nm NPs (red) and 8.93 nm NPs (blue) during the temperature increase from 30 to 800 °C.

4. NBTS-mediated assembly and light-induced disassembly of Au NPs

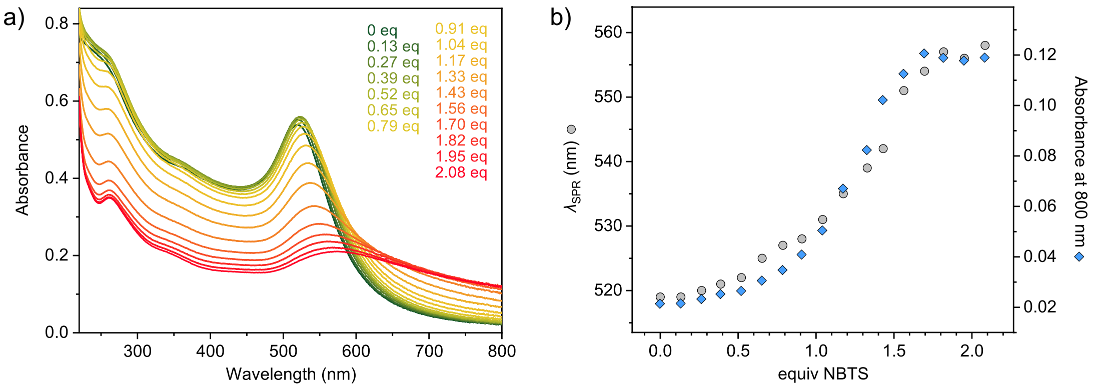

Figure S5. Self-assembly of 9.5 nm Au·TMA mediated by NBTS. (a) A series of UV/vis absorption spectra recorded during the gradual addition of NBTS to a solution of Au·TMA. (b) Gradual increase in the LSPR's wavelength of the maximum absorption (λ_{SPR}; gray markers) and the absorbance at 800 nm (blue markers) during the titration of 9.5 nm Au·TMA with NBTS ('equiv NBTS' denotes the molar ratio of the negative charges (added as NBTS) to the positive charges (i.e., the total number of NP-adsorbed TMA ligands in the titrated solution)).

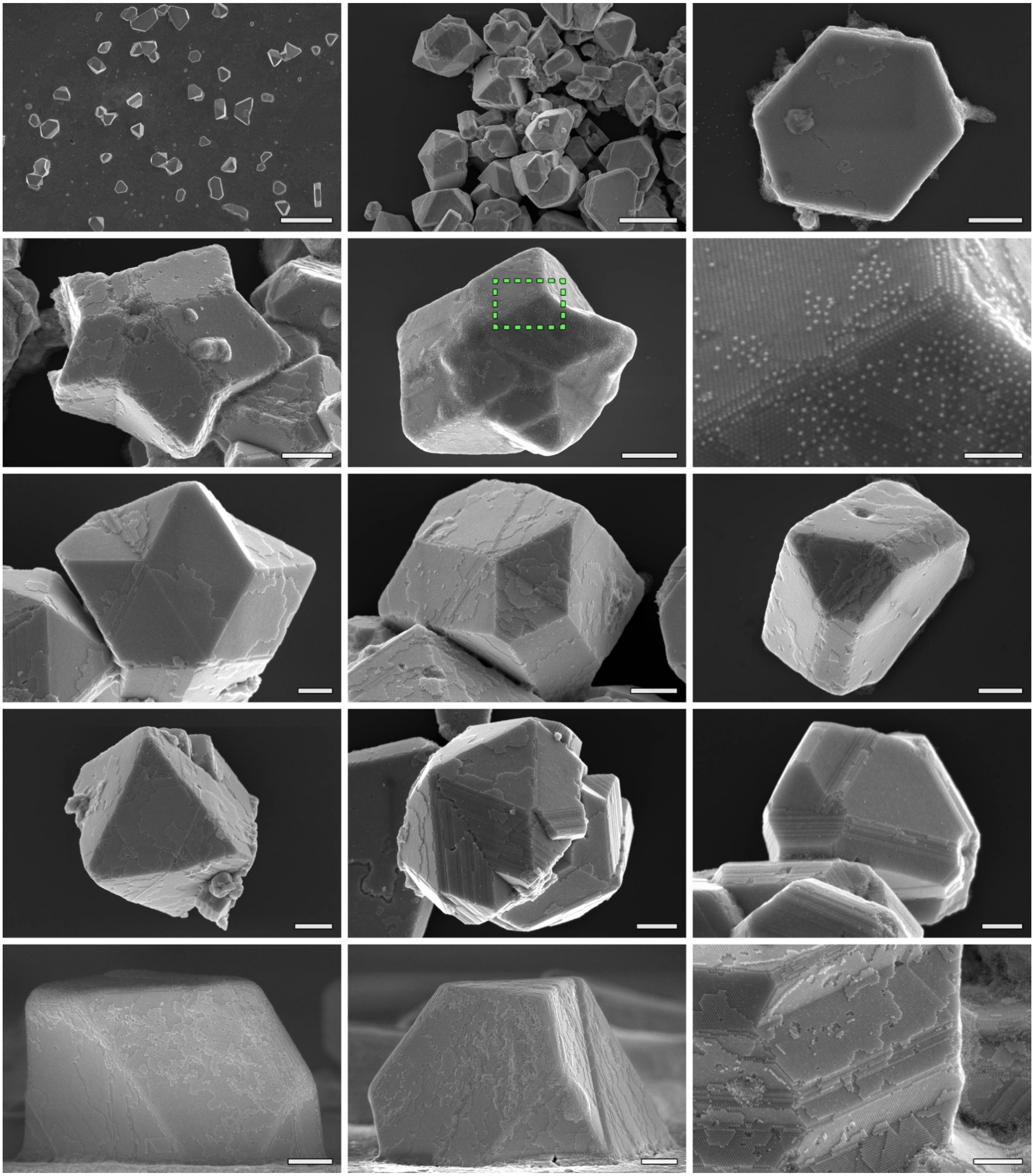

Figure S6. Additional SEM images of colloidal crystals coassembled from 9.5 nm Au·TMA and NBTS. The area denoted by the dashed green line is shown in the image on the right, which shows that extra Au·TMA (i.e., NPs whose charge was not compensated by NBTS) deposited on the surface of an Au·TMA/NBTS crystal are separated by relatively large distances, consistent with the electrostatic repulsion between them. Scalebars (left to right): *Row 1:* 5 µm, 1 µm, 500 nm; *Row 2:* 500 nm, 600 nm, 400 nm; *Row 3:* 300 nm, 1 µm, 500 nm; *Row 4:* 500 nm, 1 µm, 1 µm; *Row 5:* 200 nm, 500 nm, 200 nm.

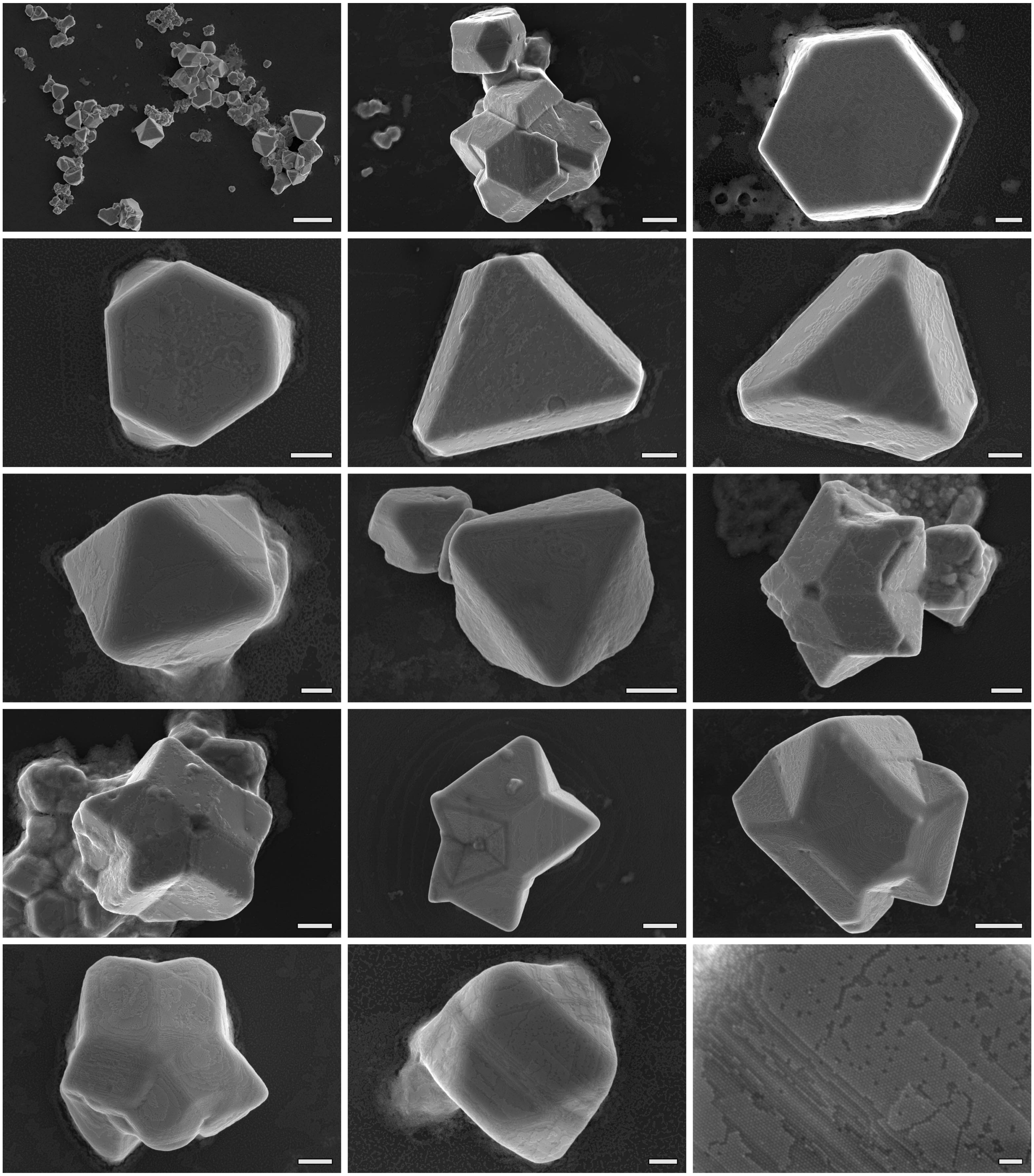

Figure S7. Representative SEM images of colloidal crystals coassembled from 5.3 nm Au·TMA and NBTS. Scalebars (left to right): *Row 1:* 10 μm, 2 μm, 500 nm; *Row 2:* 500 nm, 500 nm, 100 nm; *Row 3:* 300 nm, 500 nm, 500 nm; *Row 4:* 500 nm, 400 nm, 200 nm; *Row 5:* 500 nm, 200 nm, 50 nm.

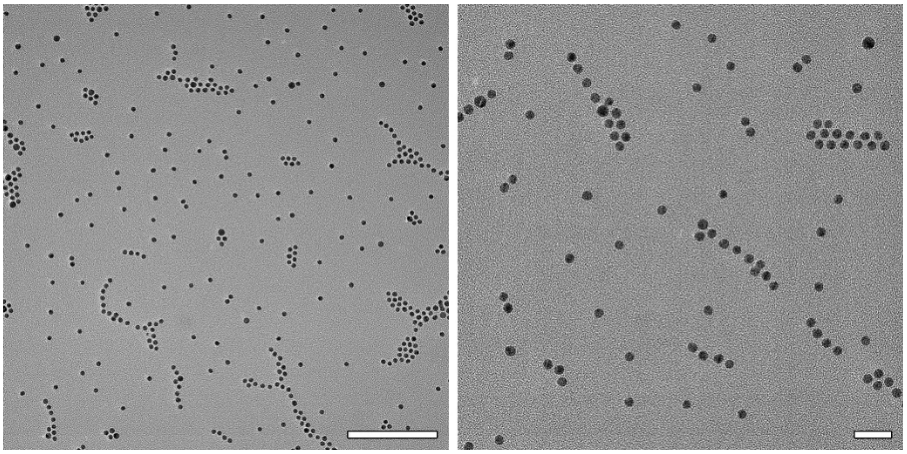

Figure S8. Representative TEM images of 5.3 nm Au·TMA after NBTS-induced assembly, followed by UV-induced disassembly. Scalebars = 100 nm (left) and 20 nm (right).

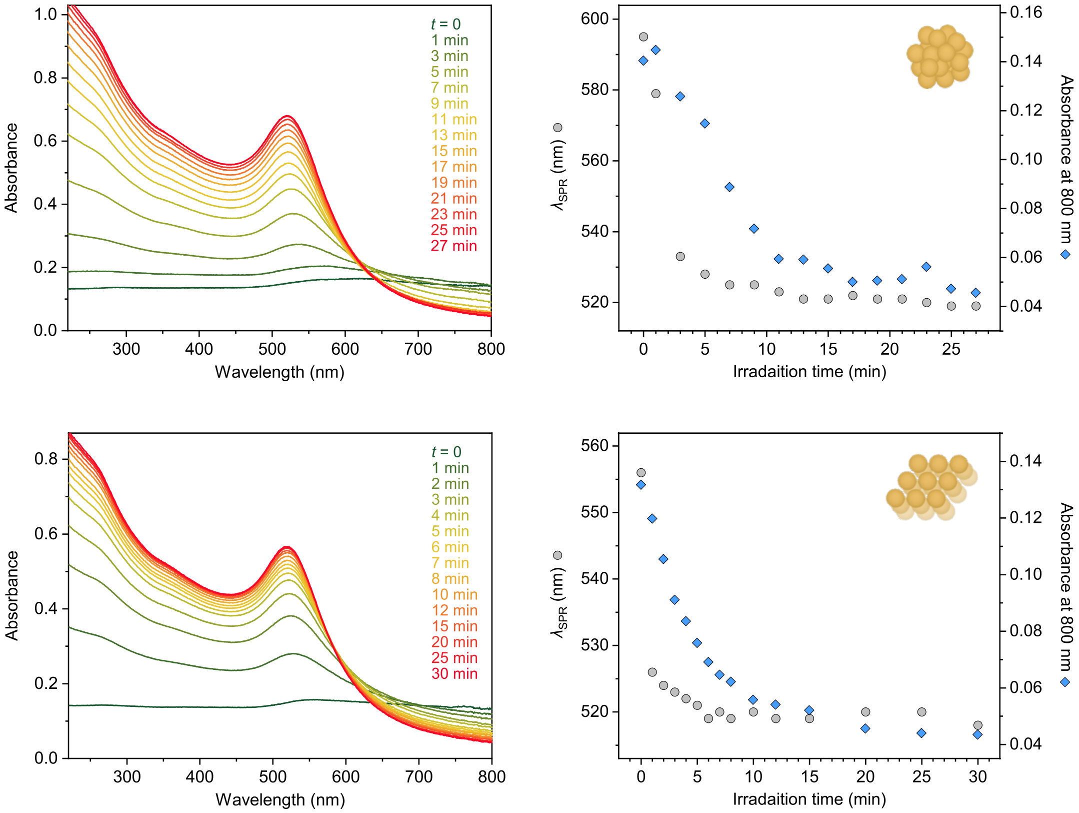

Figure S9. Comparison of the UV-induced disassembly kinetics of amorphous (top panel) vs. crystalline (bottom panel) 5.3 nm Au·TMA/NBTS aggregates.

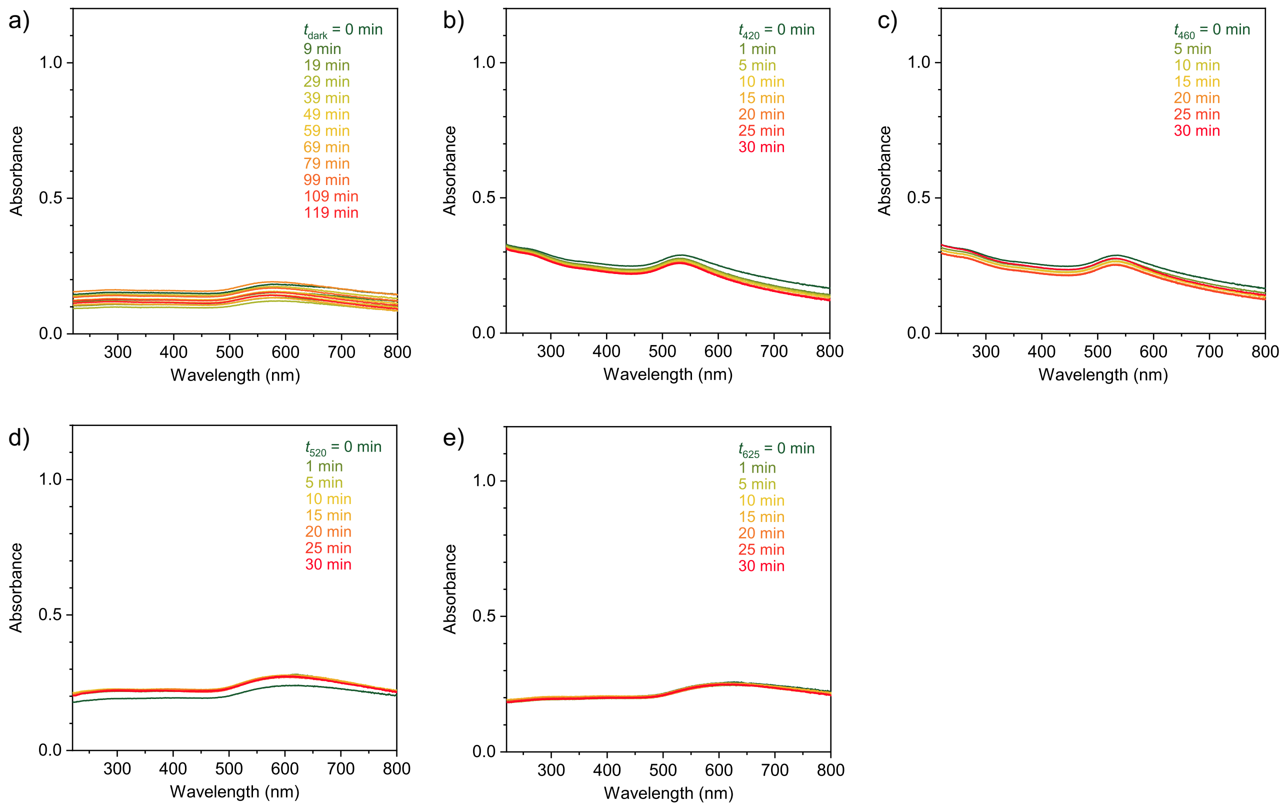

Figure S10. Control experiments showing no disassembly of 5.3 nm Au·TMA/NBTS aggregates in the dark (a) and under visible light: (b) 420 nm, (c) 460 nm, (d) 520 nm, and (e) 625 nm.

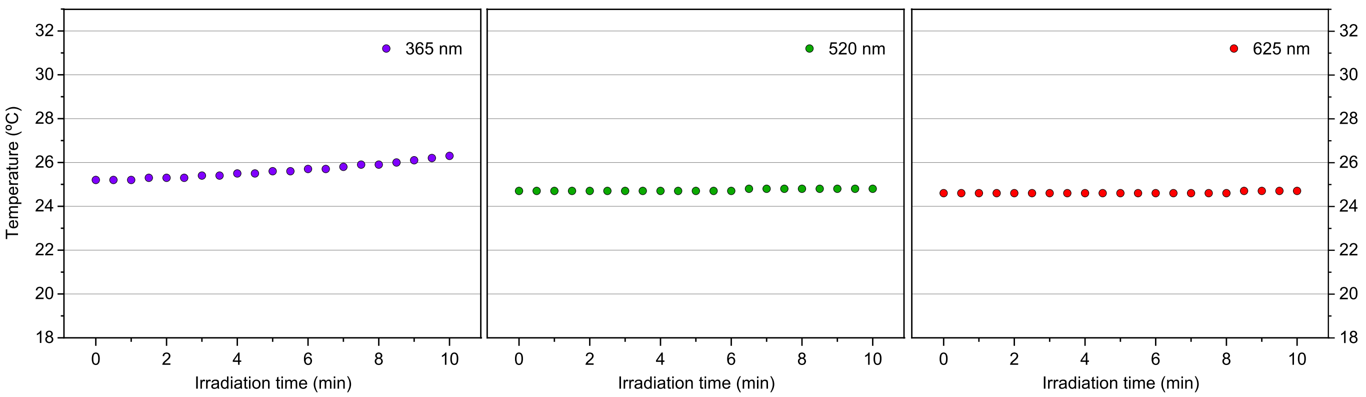

Figure S11. Effect of light irradiation at three different wavelengths on the temperature of the aqueous solution of 5.3 nm Au·TMA. The experiment was carried out at a NP concentration and with light intensities corresponding to those used in our NP assembly/disassembly experiments. Irradiation with green (525 nm) and red (625 nm) light did not induce a noticeable temperature change. Irradiation with UV (365 nm) light resulted in a small temperature increase of 1.1 ºC within 10 min; this minor change does not affect NBTS' or TOC's stability.

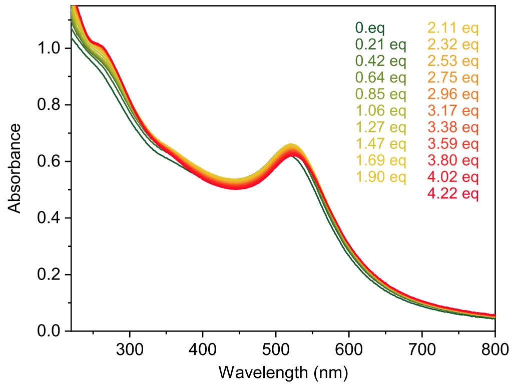

Figure S12. Control experiment showing that partially protonated NBTS (pH ≈ 5) is unable to mediate the assembly of 5.3 nm Au·TMA.

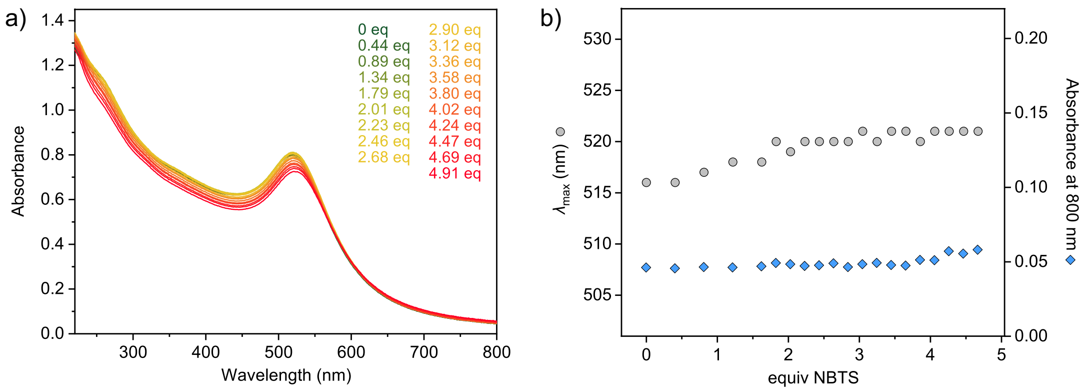

Figure S13. Control experiment showing that photocleaved NBTS (see Figure 1c for the reaction equation) is unable to mediate the assembly of 5.3 nm Au·TMA.

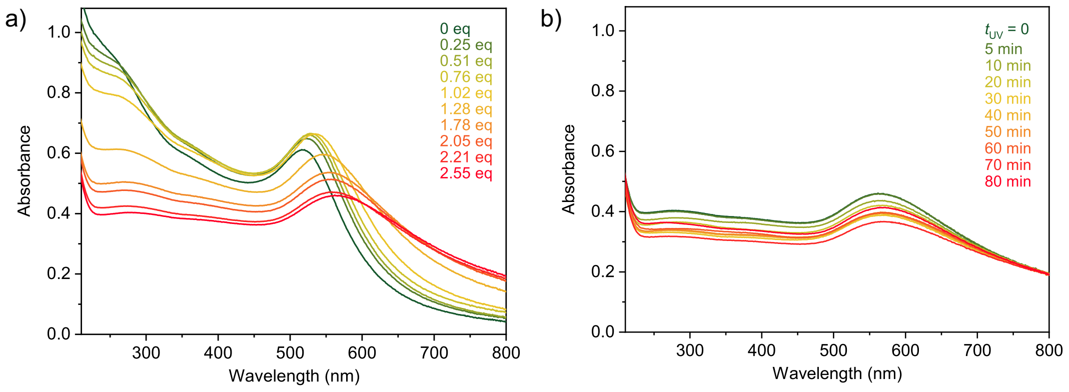

Figure S14. Control experiment showing no UV-induced disassembly of NP aggregates prepared from a non-photosensitive ionic glue (here, trisodium citrate). (a) A series of UV/vis absorption spectra recorded during the gradual addition of citrate to a solution of 5.3 nm Au·TMA. (b) A series of UV/vis spectra of Au·TMA/citrate aggregates after different times of UV irradiation.

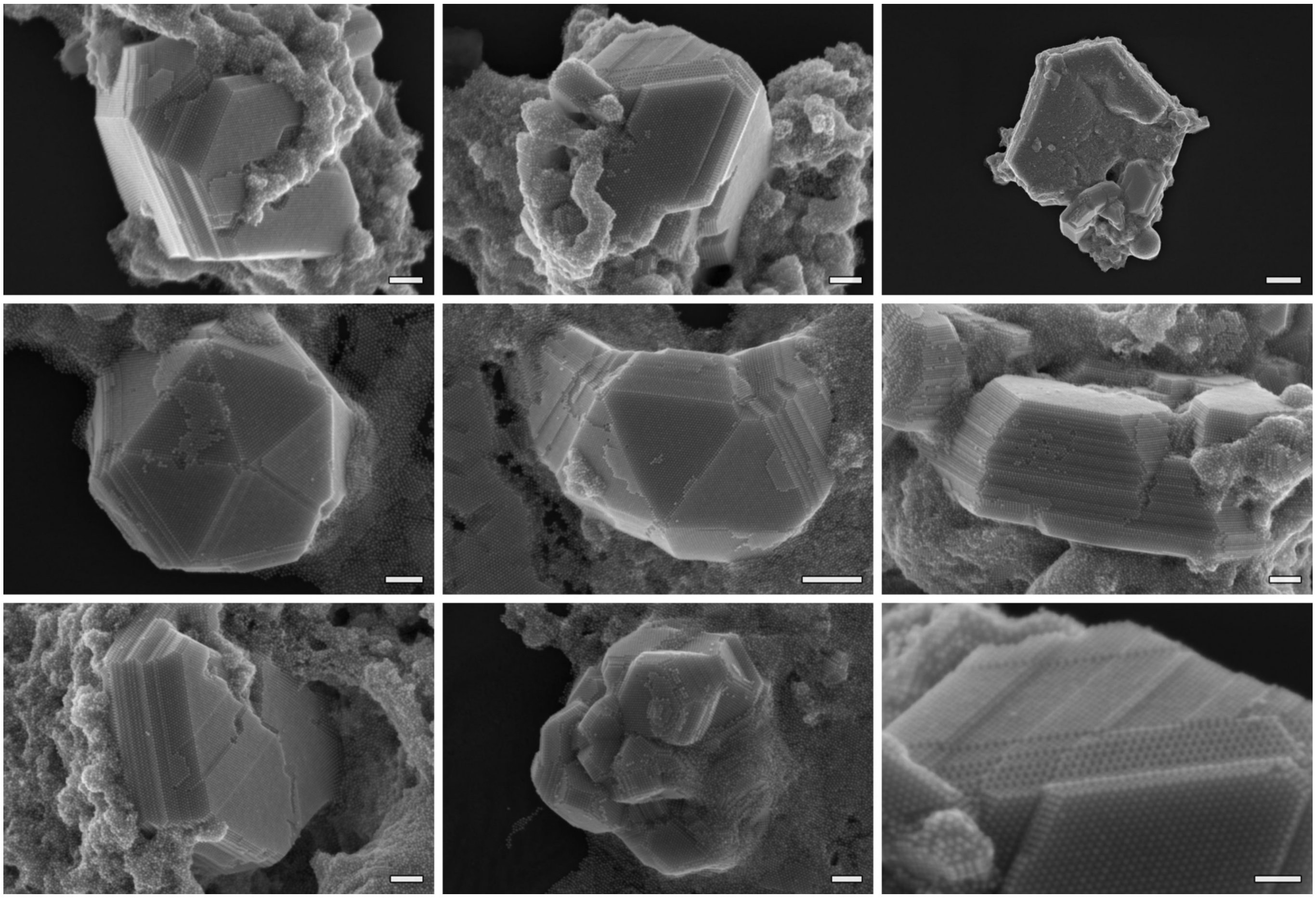

Figure S15. Representative SEM images of colloidal crystals obtained in the second cycle of NBTS-mediated self-assembly of Au·TMA. In this experiment, ~9 nm Au·TMA were first assembled into colloidal crystals as shown in Figure 3 (main text). Following UV-induced disassembly, a second aliquot of NBTS (~2 equiv) was added and the resulting amorphous aggregates were converted into crystalline ones using the ammonium carbonate method. The quality of the second-cycle crystals is lower than that of the crystals obtained in the first cycle (Figure S6) (the byproducts of the first assembly/disassembly cycle—NBDS and succinate—can interfere with crystallization in the second cycle). Scalebars (left to right): *Row 1:* 100 nm, 100 nm, 500 nm; *Row 2:* 100 nm, 200 nm, 100 nm; *Row 3:* 100 nm, 100 nm, 50 nm.

5. TOC-mediated assembly and light-induced disassembly of Au NPs

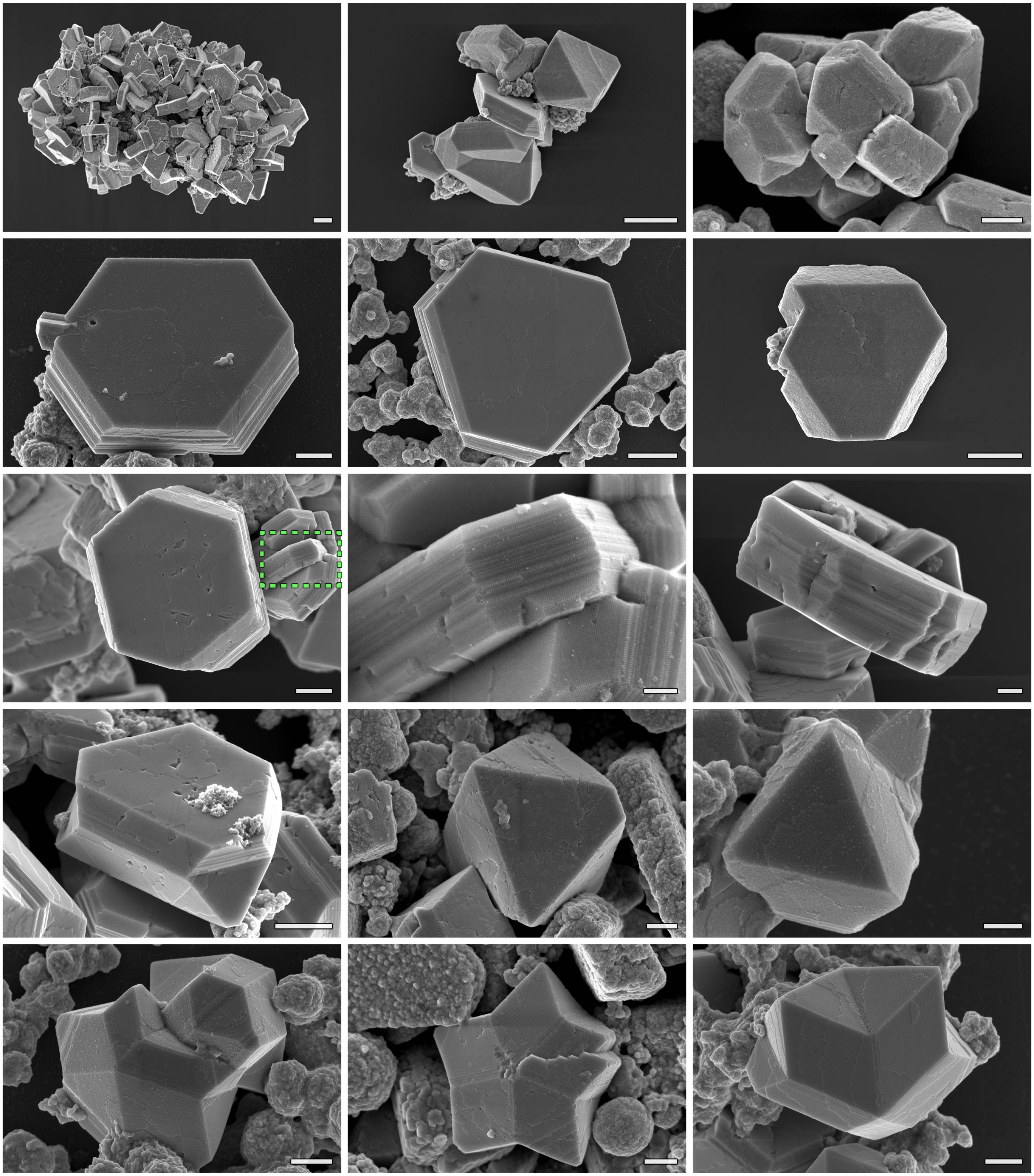

Figure S16. Additional SEM images of colloidal crystals coassembled from 5.3 nm Au·TMA and TOC (the area denoted by a dashed green line is shown on the right). Scalebars (left to right): *Row 1:* 2 μm, 2 μm, 500 nm; *Row 2:* 500 nm, 1 μm, 1 μm; *Row 3:* 500 nm, 100 nm, 200 nm; *Row 4:* 500 nm, 500 nm, 200 nm; *Row 5:* 500 nm, 200 nm, 500 nm.

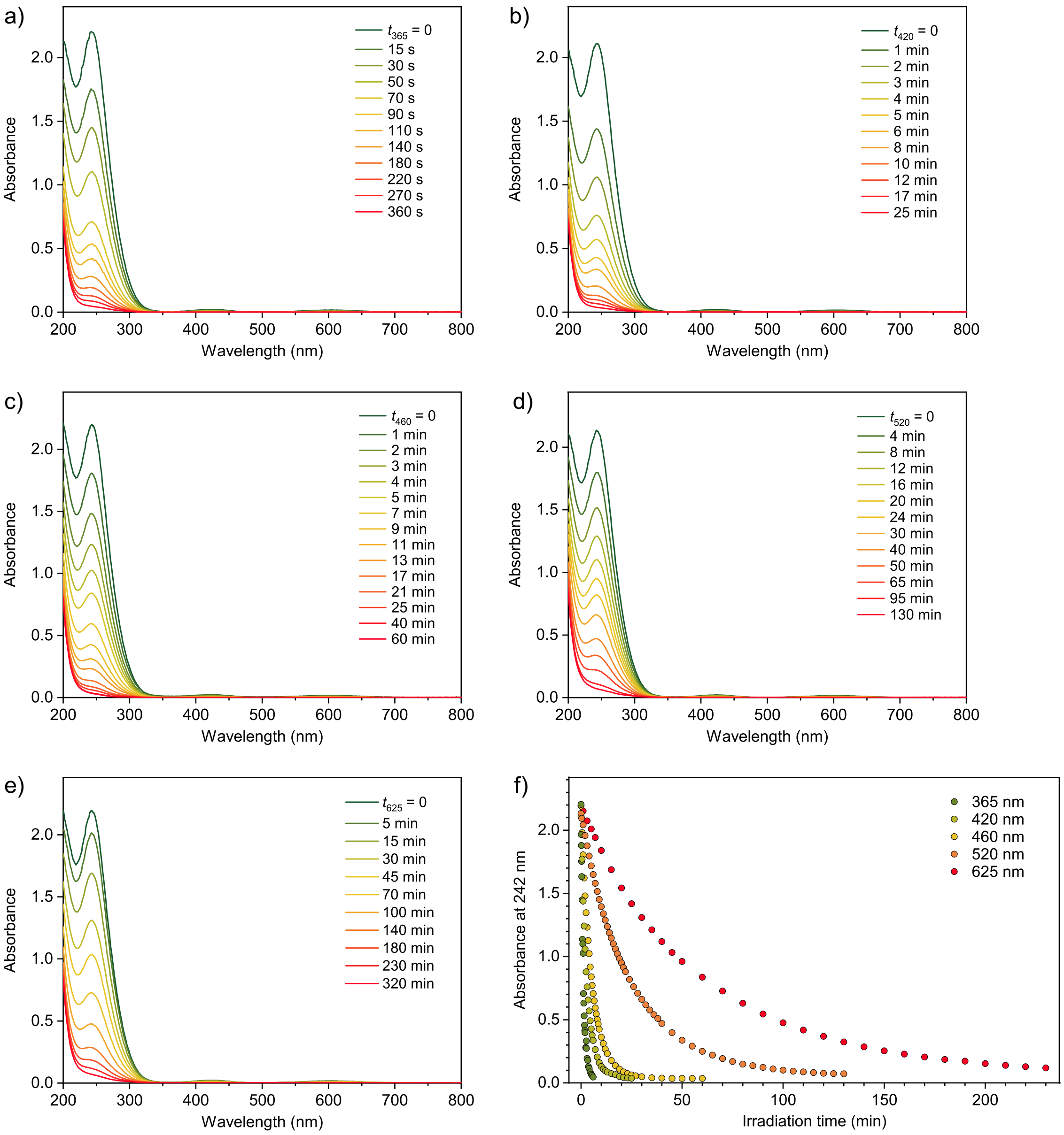

Figure S17. Following the light-induced disassembly of TOC (dissolved in water) under different wavelengths of light: (a) 365 nm, (b) 420 nm, (c) 460 nm, (d) 520 nm, and (e) 625 nm. (f) Comparison of the photodecomposition kinetics under different wavelengths of light (the intensities of all the LEDs were similar, at ~1 $mW \cdot cm^{-2}$).

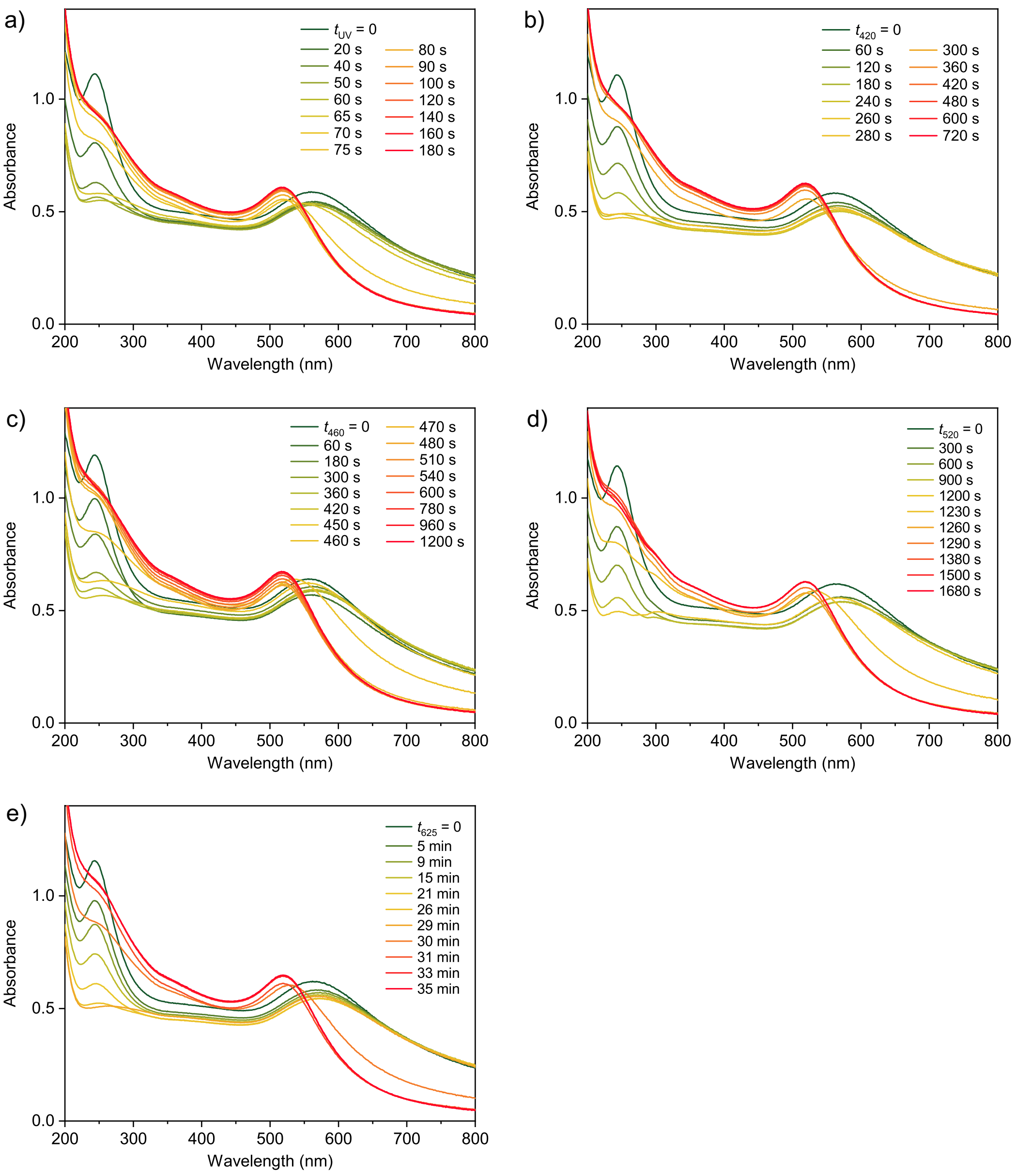

Figure S18. Wavelength-dependent decomposition kinetics of Au·TMA/TOC aggregates: (a) 365 nm, (b) 420 nm, (c) 460 nm, (d) 520 nm, and (e) 625 nm (for a summary of these results, see Figure 6f in the main text).

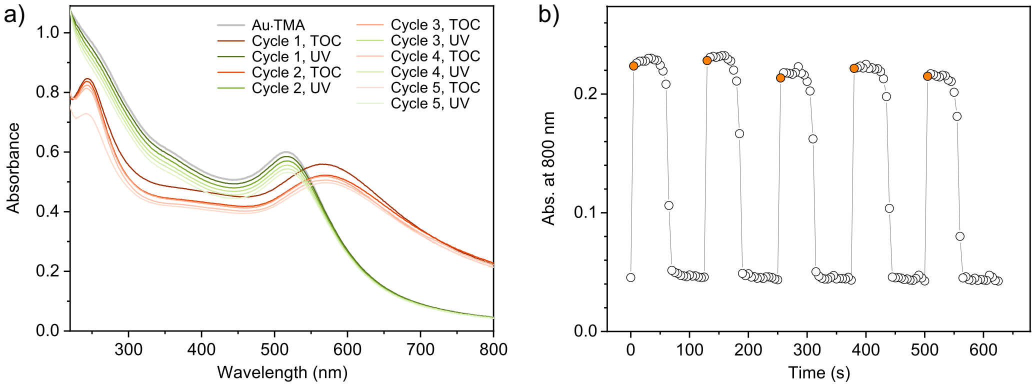

Figure S19. Reversibility of the PAG-mediated assembly–light-induced disassembly of cationic NPs. (a) Gray: The initial UV/vis spectrum of 5.3 nm Au·TMA; dark-red: 5 min after the addition of the first aliquot of TOC (~2.4 equiv); dark-green: after the subsequent UV irradiation (120 s), which completes the first assembly/disassembly cycle. The subsequent spectra follow an additional four assembly/disassembly cycles. (b) Reversible changes in the absorbance at 800 nm induced by the addition of TOC (~2.4 equiv; orange markers), followed by UV light irradiation (120 s). After adding each TOC aliquot, the system was allowed to equilibrate for 5 min.

6. Experiments with mixtures of the two PAGs

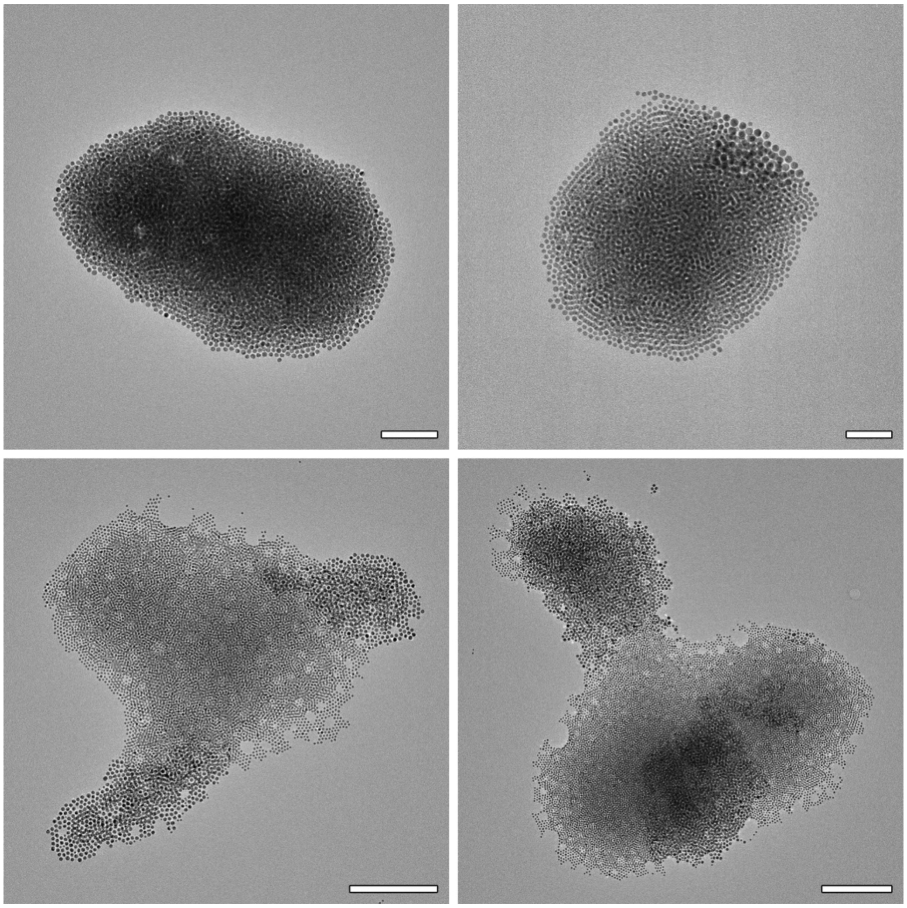

Figure S20. Representative TEM images of a sample obtained by mixing aqueous suspensions of two types of aggregates: 5.3 nm Au·TMA/TOC and 9.5 nm Au·TMA/NBTS. Clockwise starting from the top-left: i) an aggregate

consisting solely of 9.5 nm NPs; ii) an aggregate consisting of 5.3 nm NPs, with a small patch of 9.5 nm on the top-right; iii) a small aggregate of 9.5 nm NPs (top) joining a large aggregate of 5.3 nm NPs having a small patch of 9.5 nm NPs (bottom); iv) an aggregate of 5.3 nm NPs having patches of 9.5 nm NPs on the top-right and bottom-left. Scalebars (clockwise starting from the top-left: 100 nm, 50 nm, 200 nm, 200 nm.

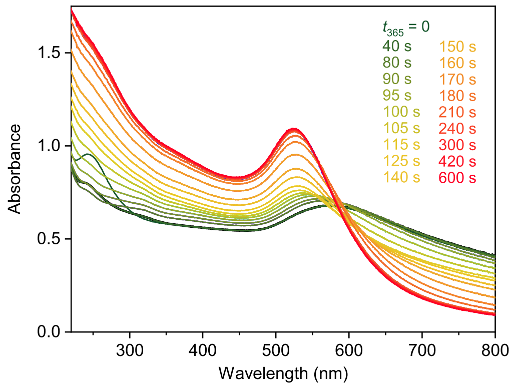

Figure S21. Changes in the UV/vis spectra of a mixture of the two types of aggregates (5.3 nm Au·TMA/TOC and 9.5 nm Au·TMA/NBTS) exposed to UV light.

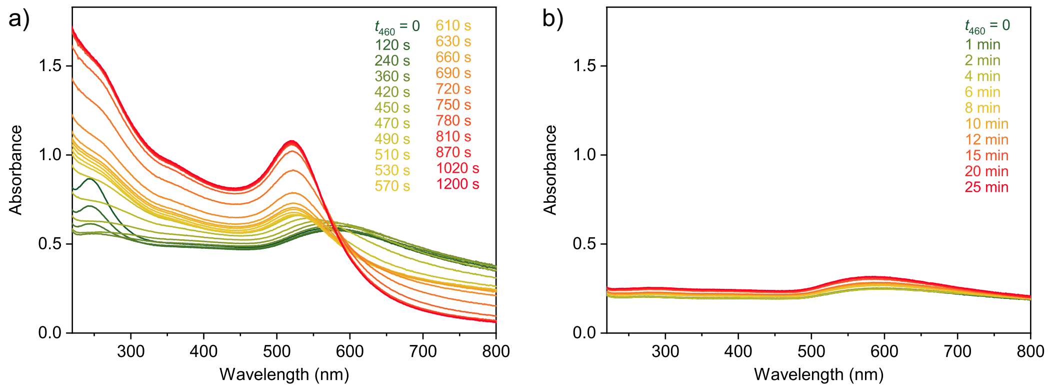

Figure S22. (a) Changes in the UV/vis spectra of a mixture of the two types of aggregates (5.3 nm Au·TMA/TOC and 9.5 nm Au·TMA/NBTS) exposed to blue light. (b) Control experiment: a lack of substantial changes in the UV/vis spectra of 9.5 nm Au·TMA/NBTS exposed to blue light.

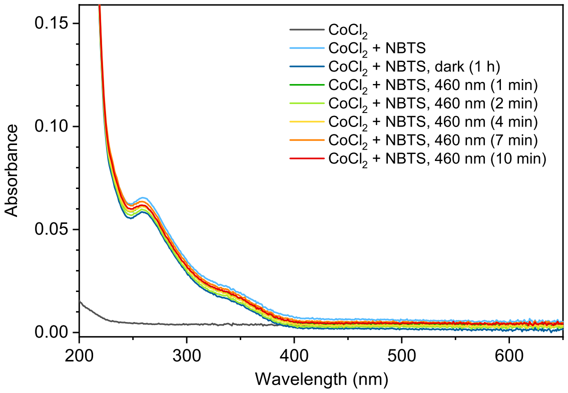

Figure S23. Control experiment demonstrating that NBTS remains stable in the presence of Co^{II} (in the dark and under blue light). The experiment was carried out in oxygenated water with a 1:1 molar ratio of NBTS to $CoCl_2$. The experiment excludes the possibility that the disassembly of the Au·TMA/NBTS aggregates in the presence of TOC exposed to visible light proceeds as a result of cobalt-catalyzed oxidation of NBTS (see Ref. 9), which would affect the characteristic UV/vis spectrum of NBTS. Instead, our results indicate that the disassembly of Au·TMA/NBTS is due to the formation of a Co^{II}/NTBS complex.

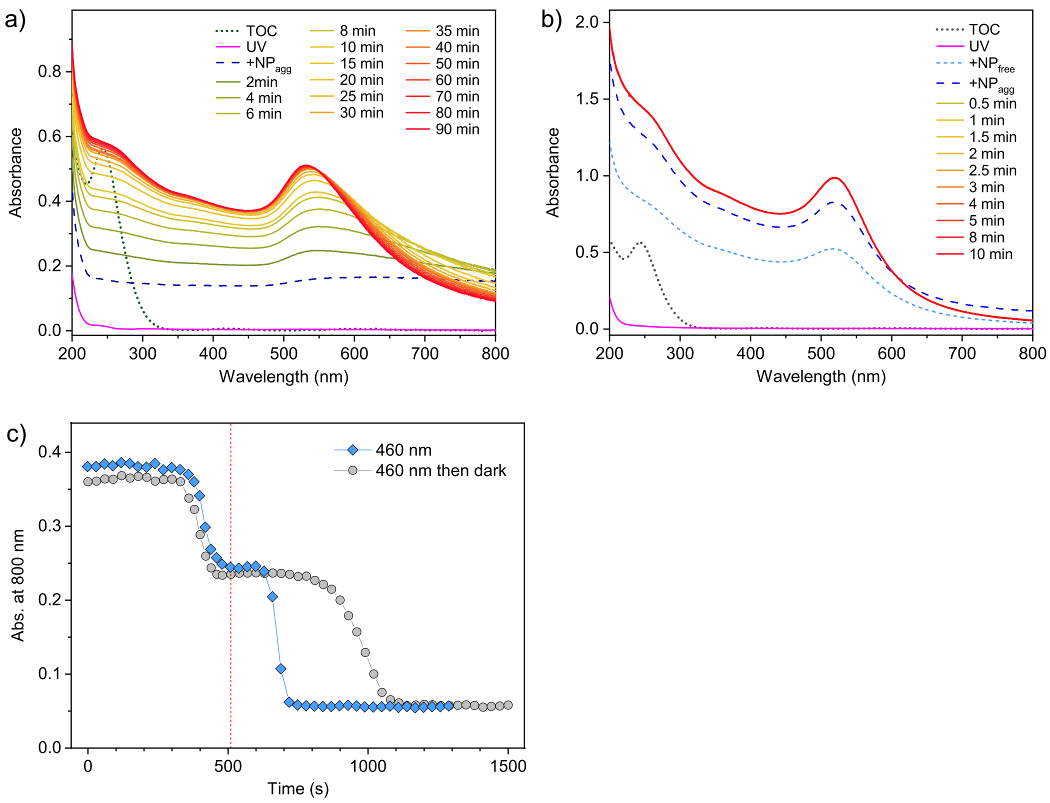

Figure S24. Control experiments with mixtures of two PAGs. (a) From the top: TOC; TOC after decomposition with UV light; after the addition 9.5 nm Au·TMA/NBTS aggregates (immediately (the dashed blue line) and after different times (green→red)). The spectra show that the disassembly of 9.5 nm Au·TMA/NBTS takes ~1 h to complete, which is attributed to the slow extraction of NBTS with Co^{II} (DOC + NBTS → Co^{II}-NBTS]$^{-}$. (b) From the top: TOC; TOC after decomposition with UV light; after the addition of 5.3 nm Au·TMA (no assembly was observed); after the subsequent addition of 9.5 nm Au·TMA/NBTS aggregates (immediately (the dashed blue line) and after different times (green →red)). The spectra show that the disassembly of 9.5 nm Au·TMA/NBTS is complete within 30 s; the

fast disassembly is attributed to breaking the electroneutrality condition (which is followed by complexation of Co^{II} by NBTS). (c) The disassembly profile of a mixture of 5.3 nm Au·TMA/TOC and 9.5 nm Au·TMA/NBTS under continuous irradiation with 460 nm light (blue markers), and under 460 nm light irradiation for the initial 510 s only (until the point denoted by the dashed red line) (gray markers).

7. Supporting references

1. West, A. P.; Smyth, N.; Kraml, C. M.; Ho, D. M.; Pascal, R. A. Synthesis, molecular structure, and properties of *in*-phosphaphanes with substituted basal aromatic rings. *J. Org. Chem.* **1993**, *58*, 3502–3506.
2. De Groot, F. M. H.; Beusker, P. H.; Scheeren, J. W. Prodrugs built as multiple self-elimination-release spacers. WO/2004/043493, May 27, 2004.
3. Bailar, J. C.; Jones, E. M.; Booth, H. S.; Grennert, M., 13. Trioxalato salts (trioxalatoaluminiate, -ferriate, -chromiate, and -cobaltiate). In *Inorganic syntheses*, 1st ed.; McGraw-Hill Book Company: 1939; pp 35–38.
4. Jong, L. I.; Abbott, N. L. Rate-dependent lowering of surface tension during transformations of water-soluble surfactants from bolaform to monomeric structures. *Langmuir* **1998**, *14*, 2235–2237.
5. Peng, S.; Lee, Y.; Wang, C.; Yin, H.; Dai, S.; Sun, S. A facile synthesis of monodisperse Au nanoparticles and their catalysis of CO oxidation. *Nano Res.* **2008**, *1*, 229–234.
6. Leff, D. V.; Ohara, P. C.; Heath, J. R.; Gelbart, W. M. Thermodynamic control of gold nanocrystal size: Experiment and theory. *J. Phys. Chem.* **1995**, *99*, 7036–7041.
7. Chu, Z.; Han, Y.; Král, P.; Klajn, R. "Precipitation on nanoparticles": Attractive intermolecular interactions stabilize specific ligand ratios on the surfaces of nanoparticles. *Angew. Chem. Int. Ed.* **2018**, *57*, 7023–7027.
8. Chu, Z.; Han, Y.; Bian, T.; De, S.; Král, P.; Klajn, R. Supramolecular control of azobenzene switching on nanoparticles. *J. Am. Chem. Soc.* **2019**, *141*, 1949–1960.
9. Li, P.; Alper, H. Cobalt-catalyzed oxidation of ethers using oxygen. *J. Mol. Catal.* **1992**, *72*, 143–152.
